# Identifying and Evaluating Salt‐Tolerant Halophytes Along a Tropical Coastal Zone: Growth Response and Desalination Potential

**DOI:** 10.1002/pei3.70072

**Published:** 2025-07-15

**Authors:** Kwabena A. Sanleri, Francis Kumi, Kwadwo K. Amoah, Solomon Amamu, Igor Luketina, Michael O. Adu

**Affiliations:** ^1^ Department of Crop Science School of Agriculture, College of Agriculture and Natural Sciences, University of Cape Coast Cape Coast Ghana; ^2^ Department of Agricultural Engineering School of Sustainable Engineering, College of Agriculture and Natural Sciences, University of Cape Coast Cape Coast Ghana; ^3^ Alchemia‐Nova GmbH Research Institute Viena Austria

**Keywords:** desalination, halophytes, phyto‐desalination, phytoremediation

## Abstract

Littoral soils along Ghana's coastal zones, hosting diverse halophytes with multiple potential applications, contain significant salt content due to seawater influence. This study identified and explored the nutritional, ecological, and medicinal significance of these halophytes, focusing on their salt tolerance and desalination abilities. Deep learning image recognition was employed to identify plant species, followed by a greenhouse experiment on five selected halophytes (*Ipomoea aquatica, Lactuca taraxacifolia, Paspalum vaginatum, Sesuvium portulacastrum,* and *Talinum triangulare* ) to assess their response to varying salt concentrations (0, 25, and 50 dS/m) and soil types (sea sand and arable soil). High salt concentrations (50 dS/m) generally reduced plant growth rates and biomass accumulation while increasing soil electrical conductivity (EC), total dissolved solids (TDS), and pH. Arable soil improved halophyte Relative Growth Rate (RGR) and performance index (PI) by 5% and 52%, respectively, compared to sea sand. *Sesuvium portulacastrum* exhibited enhanced PI at elevated salinity and demonstrated superior salt ion accumulation in roots and leaves at 50 dS/m. Both *P. vaginatum* and *S. portulacastrum* maintained the highest shoot and root dry weights under increased salinity, whereas *S. portulacastrum* significantly reduced soil EC, pH, Na, and Cl ion contents compared to other species. *Sesuvium portulacastrum* reduced several soil salinity indicators significantly compared to other species, highlighting its potential for addressing soil and water salinity issues in affected environments. This study shows the potential of Ghana's halophytes in addressing soil salinity‐related challenges.

## 1 Introduction

Soil salinity is among the most critical abiotic stressors that fundamentally disrupt agricultural productivity. Like radiation exposure, water deficit, and metal toxicity, salinity affects plant function at the molecular and ecosystem levels. The problem is expanding rapidly, with saline‐affected arable lands currently exceeding 100 million hectares globally and increasing by 40,000 ha annually (Rengasamy 2006; Shrivastava and Kumar 2015; Ahmed, Ahmed, et al. 2019). This progressive salinization induces cascading perturbations in soil physicochemical parameters and destabilizes ecological equilibrium. The implications on crop productivity are particularly severe (Hu and Schmidhalter 2002). Elevated soil salinity affects multiple biological scales through complex interactions. These range from altered gene expression patterns to compromised ecosystem functionality, ultimately reducing agricultural yields and economic viability (Singh and Chatrath 2001; Akbarimoghaddam et al. 2011; Tester and Davenport 2003). At the cellular level, salinity stress triggers a complex array of physiological responses, including ionic disequilibrium, oxidative damage, osmotic dysregulation, and impaired nutrient acquisition. The homeostasis of essential nutrients (including N, P, K, Ca, Fe, and Zn) is particularly affected (Ashraf 2004). These molecular and cellular perturbations cascade through plant developmental stages, from embryonic development through reproductive maturation. The result is both structural degradation and functional impairment across plant tissues (Liu et al. 2021; Kumar et al. 2021; Uchiyama et al. 2023).

Remedying soil salinity for crop production involves various strategies to manage, reduce, or tolerate salt levels. Soil salinity remediation could be achieved through several approaches, including leaching (Shaygan and Baumgartl 2022), improved drainage (Cuevas et al. 2019), soil amendments (Hoque et al. 2022), crop rotation (Ahmed, Qadir, et al. 2019), efficient irrigation (Wang et al. 2023), and phytoremediation, including the use of halophytic plants (Flowers and Colmer 2008). Halophytes have evolved unique physiological and biochemical mechanisms that enable them to thrive in saline environments, such as near‐shore shallows, estuaries, coastal salt marshes, inland salt lakes, and saline deserts (Flowers and Colmer 2008, 2015). Halophytes' economic viability and ability to thrive in saline soils make their cultivation advantageous. Most halophytic plants also offer multiple benefits, including providing fodder for livestock (Attia‐Ismail 2018), contributing to biodiversity and serving as habitats for various organisms (Koyro et al. 2014), and stabilizing the soil against erosion (Santos et al. 2017). Beyond their survival capabilities, halophytes play a significant role in mitigating the effects of climate change, as they are employed in soil desalination and reclamation, as well as erosion control practices (Ayyappan et al. 2016; Islam et al. 2019). Cultivating beneficial halophytes allows for the utilization of salt‐affected soils without compromising biomass or seed production and, as a result, ensures food security in saline‐affected regions. Additionally, halophytic biomass can produce biogas, providing a renewable energy source. Moreover, sustainable halophytic biomass processing offers opportunities for commercial production, including feed ingredients, food, and high‐value bioactive products, through efficient conversion and valorisation methods (Internation Energy Agency 2007).

The adaptive capabilities of halophytes extend beyond their ecological resilience, encompassing a rich ethnobotanical heritage that aligns with current economic and sustainability requirements. Indigenous communities across saline regions have historically utilized these salt‐tolerant species, developing knowledge systems for their cultivation and application. This traditional ecological knowledge now intersects with modern agricultural priorities, as halophytes demonstrate remarkable versatility in addressing multiple societal needs. Because halophytic plants can extract and sequester salts from the soil, progressively reducing soil salinity levels (Park et al. 2009), they offer a sustainable and economically viable solution for mitigating soil salinity for crop production. Thus, the salt‐tolerant mechanisms of halophytes that enable them to absorb, tolerate, or compartmentalize salts present possibilities for remediating salt‐contaminated areas for bio‐saline agricultural purposes (Manousaki and Kalogerakis 2011).

The remarkable capacity of halophytes to tolerate and remediate saline conditions presents an innovative, sustainable alternative to conventional desalination technologies (Graifenberg et al. 2003; Rabhi et al. 2009). This study employed Pl@ntNet, a digital plant identification platform, to systematically identify and characterize halophytic species in the Cape Coast littoral area. While molecular techniques such as DNA barcoding offer high accuracy, and traditional taxonomic approaches provide detailed morphological analysis, Pl@ntNet was selected for its optimal balance of accessibility, cost‐effectiveness, and reliability in field conditions. This choice addressed several practical constraints in plant identification methods. Molecular approaches require specialized laboratory infrastructure and substantial financial investment. Traditional taxonomic methods demand extensive expertise and may introduce variability in species identification across different observers. The primary objectives of this investigation were to (i) identify potential halophytic species in the Cape Coast littoral zone and characterize their ethnobotanical significance and (ii) evaluate their soil desalination efficacy across diverse soil compositions.

## 2 Materials and Methods

### 2.1 Identification of Indigenous Halophytes

The identification of halophytes was conducted along the shore of the Atlantic Ocean close to the South Campus of the University of Cape Coast in Cape Coast, Central Region, Ghana. We used the Pl@ntNet software (https://identify.plantnet.org) built on a deep‐learning model for plant image recognition (Joly et al. 2014). The Pl@ntNet is a citizen science platform for large‐scale matching learning that relies on large annotated datasets from global data collection and user uploads. The application utilizes its AI model to enable users to submit their plant observations for a prediction output. Users can then validate the prediction. The application has over 20 million observations, representing approximately 46,000 species, submitted by more than 6 million users worldwide (Lefort et al. 2024). The Pl@ntNet method was selected for its unique ability to handle large‐scale image databases and accurately identify plant species across diverse environments and morphologies. Its robustness has been demonstrated in multiple studies, such as Gaudin (2023), where it achieved reliable identification rates, particularly in ecologically diverse regions similar to our study area. Additionally, Pl@ntNet's interactive platform enables iterative verification, thereby supporting the accuracy of our identification process (Goëau et al. 2014; van der Velde et al. 2023).

Images of plants within 0–50 m of the seashore were taken. A Nikon D5600 Model 20062, SKU 5580110 digital camera was used to capture high‐resolution pictures of plants, which were submitted to the Pl@ntNet application. The application returned multiple species using a similarity search for each image. The first four matches were selected for further investigation. Each matched plant was used as a search term in plant repositories, including Useful Tropical Plants (https://tropical.theferns.info), World Flora Online (https://worldfloraonline.org), Global Biodiversity Information Facility (https://gbif.org), and JSTOR Global Plants (https://plant.jstor.org), to verify characteristics pertinent to halophytes and examine their individual properties.

Each matched plant was examined based on its growth patterns, particularly focusing on its growth behavior (whether a shrub, vine, herb, or tree) compared to that observed in the images. Additionally, their ecological preferences were assessed to determine whether they could survive and thrive in saline environments, such as seashores. Furthermore, their distribution was evaluated to determine whether they are found in the tropics, and more specifically, in Ghana. Plants that met all these criteria were identified as halophytes. The identified halophytes were subsequently categorized based on their specific attributes, such as their growth patterns, propagation methods, life cycles, and botanical properties, into distinct groups, including succulents, shrubs, vines, and herbaceous plants. Moreover, as mentioned earlier, the practical applications and significance of the plant in food, feed, medicine, and ornamental uses were carefully assessed through the plant repositories. Criteria for selecting halophytes for subsequent experiments included a combination of desirable attributes, such as simple propagation methods (preferably propagated by cuttings), high survival rates, short life cycles, and proven beneficial uses. *Ipomoea aquatica* , *Lactuca taraxacifolia*, *Paspalum vaginatum* , and *Sesuvium portulacastrum* were selected for the study, with *Talinum triangulare* , a locally beneficial MIO halophyte (plants that grow in habitats with low levels of salinity, typically < 0.5% NaCl) (Bamidele et al. 2007), used as a control crop.

### 2.2 Assessing the Desalination Ability of Identified Plants

#### 2.2.1 Experimental Designs and Treatments

Following the identification, a greenhouse study was conducted at the A. G. Carson Technology Centre, School of Agriculture, University of Cape Coast, Ghana. The Centre lies between longitude 1°15 W and latitude 5°07 N at 1.1 m above sea level in the Cape Coast municipality in the Coastal Savanna Agroecological zone (Ampofo 2006). A 5 × 3 × 2 factorial design was laid out in a completely randomized design. The factors were halophyte species, salt concentrations, and soil types (Table 1). Each treatment was replicated four times.

**TABLE 1:** Experimental design factors: Halophyte species (*n* = 5), salt treatment (*n* = 3), and soil types (*n* = 2) used in the factorial greenhouse experiment.

Factors		
Halophyte species	Salt concentrations (dS/m)	Soil types
*Ipomoea aquatica* (IA)	0	Arable soil (UCC)
*Lactuca taraxacifolia* (LT)	25	Sea sand
*Paspalum vaginatum* (PV)	50	
*Sesuvium portulacastrum* (SP)		
*Talinum triangulare* (TT)		

#### 2.2.2 Soil Preparation and Physicochemical Analysis

Samples of both arable soil and sea sand were collected and analyzed to ensure proper characterization and suitability for the study. The arable soil used in the experiment was obtained from an area adjacent to the Teaching and Research Farm, UCC. It was sampled at a 0–15 cm depth, considering the topsoil layer where significant plant‐root interactions occur. The soil belonged to the *Benya* series, classified as a sandy clay loam, specifically identified as a member of the *Edina‐Benya association* (Asamoah 1973). It demonstrated characteristics of haplic acrisol, a soil type commonly found in Ghana's coastal savanna agroecological zone.

Similarly, sea sand was gathered from the seashore, meticulously washed to eliminate impurities and remove salt content, and left to air dry naturally. Kumi et al. (2023) have described the methods used in determining soil properties. The field capacities of the two soil types were determined as described by Cassel and Nielsen (1986) and Akhter et al. (2004) for arable soil (UCC) and sea sand, respectively. Table 2 shows the basic physicochemical characteristics of the soils.

**TABLE 2:** Physicochemical properties of the soils.

Parameter	Unit	Soil type	
		SEA	UCC
Clay		—	33
Silt		—	5
Sand	%	100	63
OC		0.42	1.40
N		0.02	0.06
P		0.65	2.25
K		0.97	1.46
Ca	mg kg^−1^	2.17	4.68
Mg		1.04	2.46
BD	g cm^−3^	1.80	1.35
pH	—	6.41	5.71

#### 2.2.3 Planting Material Preparation and Planting

Due to their low survival rates, the rhizomes of *Lactuca taraxacifolia* and stem cuttings of *Ipomoea aquatica* were initially nursed on beds to encourage shoot sprouting. After 2 weeks, the resulting shoots were transplanted into the pots. On the other hand, *Paspalum vaginatum* , *Sesuvium portulacastrum* , and *Talinum triangulare* were directly planted into the pots using stem cuttings. Cuttings measuring approximately 15 cm long were planted into the pots on the same day. A pot was dedicated to a single halophyte cutting or sprout. Each halophyte species was planted in 24 pots, with 12 in arable soil and 12 in sea sand, resulting in 120 experimental units. The plants were irrigated three times weekly at 100% field capacity to ensure plant establishment and were grown for 2 weeks before the imposition of salt treatment.

#### 2.2.4 Simulating Seawater Conditions and Preparing Irrigation Water

Local salt crystals were sourced from a local market in Elmina, Ghana. Salt crystals were added to deionized water at approximately 17.5 and 35 g/L, respectively, and stirred using a rod to prepare saline solutions with experimental salinity levels of 25 and 50 dS/m, simulating seawater conditions (Doe 1994; National Oceanic and Atmospheric Administration 2023). These salinity levels were selected based on seawater's half and full strength. The mixing process involved adding the calculated amounts of sea salt to deionized water in large containers and using a rod for at least 30 min to ensure thorough dissolution. Electrical conductivity (EC) was measured with an EC meter to confirm the desired salinity levels of 25 and 50 dS/m, and adjustments were made by adding small amounts of sea salt crystals or deionized water as necessary. After allowing the solutions to sit and reach temperature equilibrium, final EC measurements were taken to verify stability. Prepared irrigation water was stored in clean, labeled containers to prevent contamination and evaporation. We monitored pH using a pH meter (Yinnik Multifunction Meter; BLE‐C600), adjusting with diluted HCl or NaOH to maintain a pH of around 7.5. The irrigation water without added salt was the 0 dS/m control treatment. The salt concentrations were applied at 350 mL per pot 2 weeks after planting. Salt treatments were applied uniformly across all species to standardize experimental conditions and eliminate timing as a variable. This allows direct comparison of species‐specific salt tolerance responses. After imposing salt concentration, normal irrigation water was applied weekly at 60% field capacity (FC). This irrigation frequency and volume were selected based on preliminary trials that demonstrated optimal plant growth at 60% FC while preventing leaching of the salt treatment applied.

### 2.3 Data Collection

Plant height was measured using a measuring tape from the soil's surface to the plant's tip before applying the salt concentrations and bi‐weekly thereafter for ten (10) weeks. Due to the diverse plant types and growth patterns of the selected halophytes, we opted for relative growth measurements, as they enable normalizing growth to the size of the plant and facilitate the comparison of growth rates across different species and concentrations. Relative Growth Rate (RGR) was calculated from plant height for each plant using Equation (1).

(1) $$\mathrm{RGR}=\left(\ln\;W2-\ln\;W1\right)/\left(T2-T1\right)$$

where W2 represents the final plant height, W1 represents the initial plant height before salt concentration application, T2 represents the final week of data taking (i.e., 10 weeks after salt imposition), and T1 represents the first week of data taking (before salt treatment application) (Simpson et al. 2018).

Harvesting was conducted ten (10) weeks after the imposition of the salt concentrations to standardize the data collection process and facilitate direct comparisons among the selected halophytes. Although the selected plants had different natural maturity times, this study focused not on their maturity or yield at their natural harvest stage but on their growth responses and salt tolerance under controlled conditions. The shoots were cut at the soil surface, bagged, and conveyed to the lab for further analysis. The roots were excavated and washed under high‐pressure water to remove the soil and debris. Weights were determined for fresh shoots and roots. Shoots and roots were then oven‐dried at 65°C for 72 h until constant weight was achieved to obtain dry weights (Muchate et al. 2016). Root‐to‐shoot ratios were calculated using dry weight measurements (dry root weight/dry shoot weight).

Soil and plant samples were analyzed for Na and Cl contents. Samples were air‐dried, powdered gently with a wooden mallet, and passed through a 2 mm sieve. Soil and plant Na and Cl contents were determined using the Inductively Coupled Plasma Optical Emission Spectrometry (ICP‐OES) method described by Sikora and Moore (2014). The soil samples' EC, TDS, and pH were estimated using a 1:2 soil‐to‐water‐by‐volume ratio, as Adu et al. (2017) indicated using a calibrated and validated Yinnik Multifunction Meter (BLE‐C600).

### 2.4 Data Analysis

Descriptive statistics were generated to summarize the data. Assumption checks were conducted for all response variables. Shapiro–Wilk's test was conducted to determine data normality, and Levene's tests were performed to verify homogeneity of variance across treatment groups. All variables met ANOVA assumptions (*p* > 0.05). Analysis of variances (ANOVA) was conducted to determine whether the growth of halophytes was significantly affected by soil type and salinity level and whether there were significant interactions. The ANOVA was also conducted to determine if there were significant differences in salt tolerance, growth, and biomass accumulation among the treatments. The factors for the ANOVA were halophyte species, soil type, and salt concentration level. Where ANOVA detected significant differences, the Least Significant Difference (LSD) test at a 5% level was used to separate means. All statistical analyses were performed using the R software program version 4.2.1 (R. Core Team 2022). Graphs were created using the ggplot2, dplyr, ggh4x, and extrafont packages and libraries in R‐Studio (R. Core Team 2022 version 4.2.1).

## 3 Results

### 3.1 Types of Halophytes in the Study Location

**FIGURE 1:**
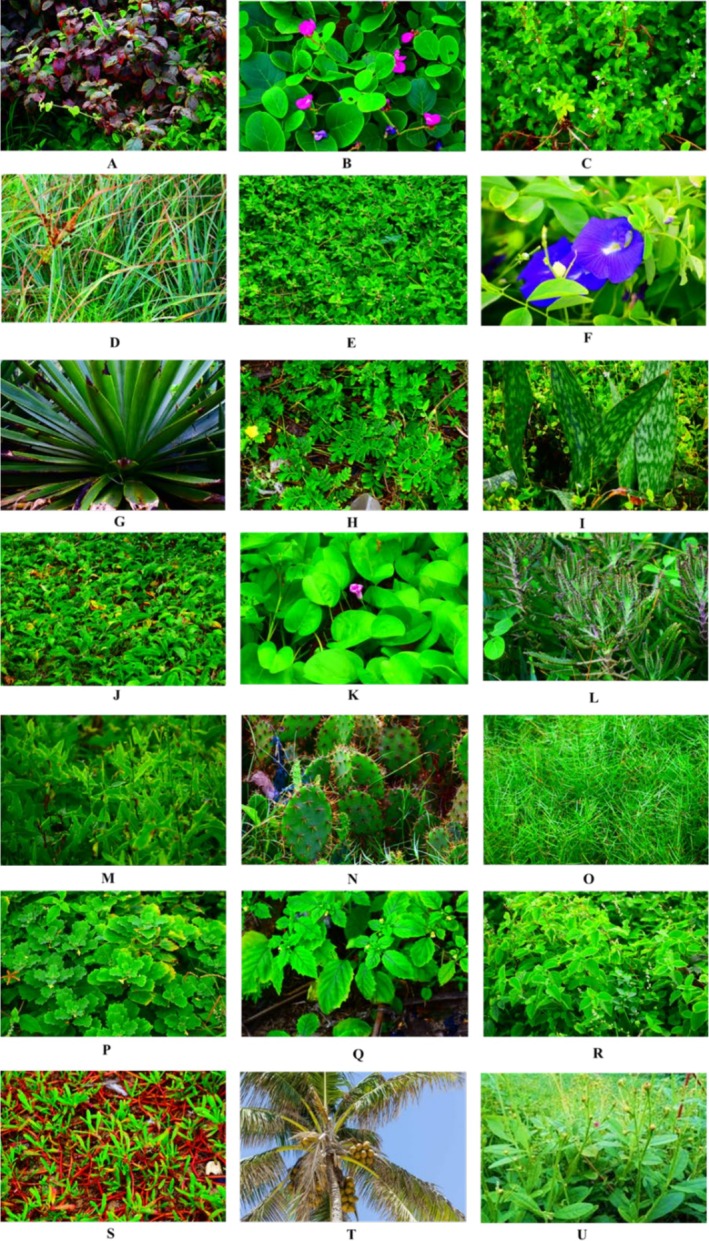
Halophytes identified in this study. (A) *Amaranthus* spp. (B) *Canavalia rosea*, (C) *Capraria biflora* (D) *Cyperus ligularis*, (E) *Euphorbia albomarginata*, (F) *Clitoria ternatea*, (G) *Furcarear cabuya*, (H) *Indogofera spicata*, (I) *Sansevieria masoniana*, (J) *Ipomoea aquatica*, (K) *Ipomoea asarifolia*, (L) *Kalanchoe daigremontiana*, (M) *Lactuca taraxacifolia*, (N) *Opuntia* spp., (O) *Paspalum vaginatum*, (P) *Pedalium murex*, (Q) *Physalis spp*, (R) *Pupalia lappacea*, (S) *Sesuvium portulacastrum*, (T) *Cocos nusifera*, (U) *Talinum triangulare*.

Twenty (20) halophytes were identified (Figure 1). Nearly half (48%) of the identified halophytes were herbaceous plants. Shrubs and succulent plants each constituted 14% of the identified plants. Also, two‐thirds of the halophytes were perennials, while the remaining third were annuals. Regarding growth habits, most (71%) of the halophytes are upright plants, 14% are vines, and the remaining are palms or prostrate.

### 3.2 Relative Growth Rate (RGR), Performance Index (PI), and Biomass Accumulation

**FIGURE 2:**
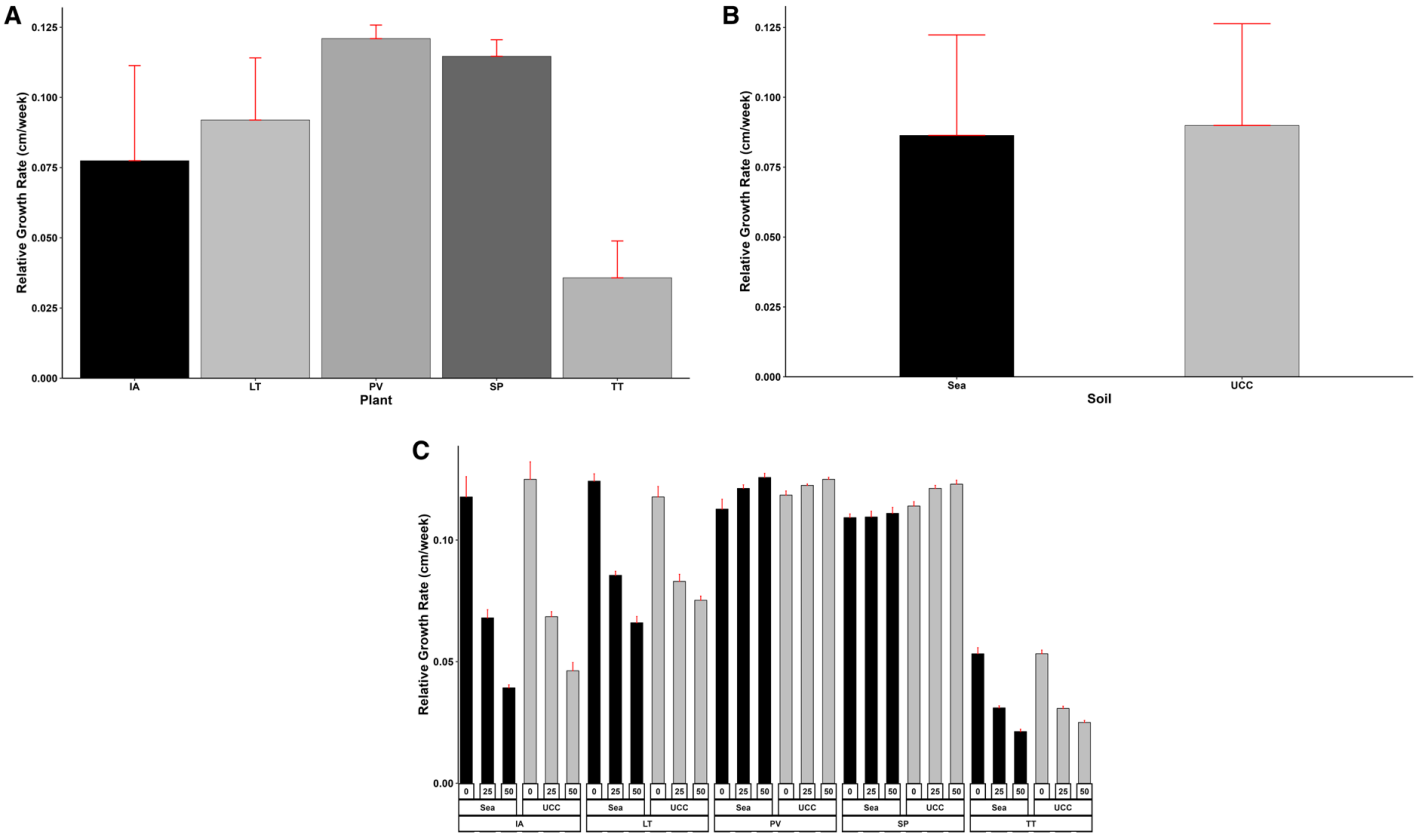
Relative growth rate of (A) Plant types (IA—*I. aquatica*, LT—*L*. *taraxacifoliar*, PV—*P. vaginatum*, SP— *S. portulacastrum,* and TT— *T. triangulare* ), (B) Soil types (UCC, Sea), and (C) Combined plant and soil types under salt concentrations (0, 25, 50 dS/m). Values represent means ± SD.

The halophyte species, soil types, and salt concentrations had a significant (*p* < 0.01) impact on the relative growth rate of the halophytes. Generally, RGR decreased with increasing salt concentrations except for *P. vaginatum* and *S. portulacastrum*, which exhibited increased RGR with increasing salt concentrations. *Paspalum vaginatum* and *S. portulacastrum* (0.12 cm/week, each) had the highest RGR at increased salt concentrations (i.e., at 25 and 50 dS/m salt concentrations), which were 50%, 100%, and 300% faster compared to *L. taraxacifoliar*, *I. aquatica* , and *T. triangulare*, respectively (Figure 2A). Plants in UCC soil (0.03 to 0.13 cm/week) generally resulted in RGR 5% higher than those in sea sand (0.02 to 0.13 cm/week) (Figure 2B). Similarly, the increasing salt concentrations reduced the RGR of the halophytes by 25% and 38% at 25 and 50 dS/m, respectively. In addition, the interactions between the halophyte species, soil types, and salt concentrations were highly significant (*p* < 0.001). For instance, *P. vaginatum* in sea sand at 50 dS/m salt concentration attained the fastest growth rate of 0.13 cm/week, whereas *T. triangulare* in sea sand at 50 dS/m salt concentration attained the slowest growth rate of 0.02 cm/week. The former was 550% faster than the latter (Figure 2C).

**FIGURE 3:**
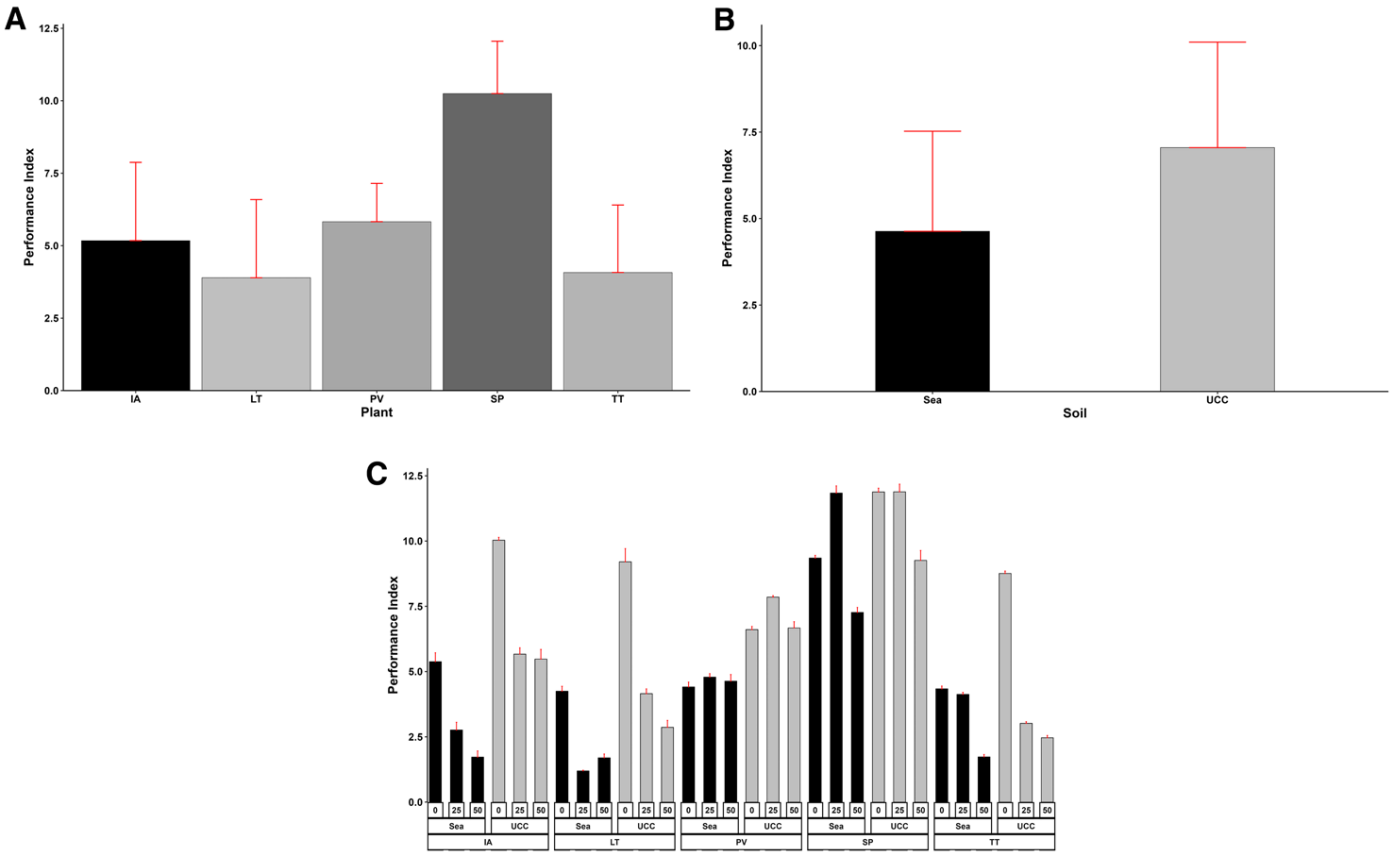
Performance Index of (A) Plant types (IA—*I. aquatica*, LT—*L*. *taraxacifoliar*, PV—*P. vaginatum*, SP— *S. portulacastrum,* and TT— *T. triangulare* ), (B) Soil types (UCC, Sea), and (C) Combined plant and soil types under salt concentrations (0, 25, 50 dS/m). Values represent means ± SD.

The performance index varied greatly among the plant species, salt concentrations, and soil types (*p* < 0.001). The interactions between the three factors were also significant (*p* < 0.001). *Sesuvium portulacastrum* attained the highest performance index of 10.25, followed by *P. vaginatum* (5.83), *I. aquatica* (5.17), *T. triangulare* (4.07), and *L*. *taraxacifoliar* (3.89) (Figure 3A). Generally, the performance index tended to reduce with increasing salt concentrations, recording a mean of 7.42 at 0 dS/m, which is 30% and 69% higher compared to means at 25 dS/m (5.73) and 50 dS/m (4.38), respectively. However, *S. portulacastrum* recorded 15% and 3% higher PI at 25 and 50 dS/m salt concentrations, respectively, while *P. vaginatum* recorded 12% higher PI at 25 dS/m and 28% lower at 50 dS/m. Also, UCC soil improved the performance index of the halophytes by 52% (Figure 3B). In UCC soil at 25 dS/m salt concentration, *S. portulacastrum* obtained the highest performance index of 11.89, followed by the same halophyte and the same soil at 0 dS/m salt concentration (11.88), whereas *L*. *taraxacifoliar* in sea sand at 25 dS/m salt concentration recorded the lowest performance index of 1.2 (Figure 3C).

Both shoot and root dry weights were significantly (*p* < 0.001) affected by the plant species, soil types, and salt concentrations, as well as their interactions (Table 3). Generally, UCC soil significantly increased shoot dry weight (SDW) by 10.35 g and root dry weight (RDW) by 0.87 g compared to sea sand. Similarly, SDW increased by 0.91 and 0.84 g in the 50 and 25 dS/m salt concentrations, respectively. Meanwhile, RDW was reduced in the 50 and 25 dS/m salt concentrations by 0.41 and 0.14 g, respectively. *Paspalum vaginatum* accumulated the most SDW of 16.47 g, followed by *S. portulacastrum* (12.61 g), *I. aquatica* (5.23 g), *T. triangulare* (2.91 g), and *L. taraxacifoliar* (2.55 g). For RDW, *P. vaginatum* recorded the highest of 2.76 g, while *T. triangulare* had 2.08 g, followed by *L. taraxacifoliar* (1.72 g), *I. aquatica* (1.07 g), and *S. portulacastrum* (0.99 g) (Table 3).

**TABLE 3:** Effects of salt concentrations and soil type on SDW (shoot dry weight, g), RDW (root dry weight, g), and R/S Ratio (root to shoot ratio) of selected halophytes.

Plant	Salt	SDW (G)		RDW (G)		R/S ratio	
		Sea	UCC	Sea	UCC	Sea	UCC
IA	0	1.70 ± 0.15	10.61 ± 0.43	1.17 ± 0.05	1.80 ± 0.05	0.69 ± 0.06	0.17 ± 0.00
	25	1.19 ± 0.04	8.95 ± 0.54	0.70 ± 0.04	1.28 ± 0.07	0.59 ± 0.03	0.14 ± 0.00
	50	0.97 ± 0.09	7.96 ± 0.29	0.43 ± 0.03	1.05 ± 0.06	0.45 ± 0.05	0.14 ± 0.01
LSD_0.05_		0.18	0.56	0.08	0.12	0.1	0.01
LT	0	0.59 ± 0.03	5.59 ± 0.21	0.97 ± 0.08	3.50 ± 0.16	1.66 ± 0.11	0.63 ± 0.04
	25	0.38 ± 0.03	4.65 ± 0.30	0.57 ± 0.03	2.90 ± 0.15	1.50 ± 0.14	0.63 ± 0.05
	50	0.28 ± 0.02	3.81 ± 0.20	0.39 ± 0.02	2.00 ± 0.11	1.39 ± 0.10	0.53 ± 0.05
LSD_0.05_		0.04	0.04	0.1	0.19	0.21	0.08
PV	0	3.47 ± 0.27	25.45 ± 2.42	1.44 ± 0.20	2.38 ± 0.15	0.42 ± 0.07	0.09 ± 0.01
	25	5.32 ± 0.25	28.93 ± 1.72	2.61 ± 0.20	3.50 ± 0.17	0.49 ± 0.04	0.12 ± 0.01
	50	5.69 ± 0.12	29.99 ± 2.15	2.62 ± 0.14	4.03 ± 0.23	0.46 ± 0.01	0.13 ± 0.01
LSD_0.05_		0.46	3.92	0.33	0.35	0.08	0.02
SP	0	4.46 ± 0.32	14.62 ± 0.25	0.61 ± 0.04	1.40 ± 0.10	0.14 ± 0.01	0.10 ± 0.01
	25	6.57 ± 0.28	20.23 ± 0.37	0.79 ± 0.03	1.27 ± 0.14	0.12 ± 0.00	0.06 ± 0.01
	50	6.58 ± 0.31	23.20 ± 1.41	0.68 ± 0.03	1.22 ± 0.04	0.11 ± 0.01	0.06 ± 0.01
LSD_0.05_		0.39	1.61	0.06	0.19	0.02	0.01
TT	0	1.93 ± 0.19	5.27 ± 0.30	2.73 ± 0.15	3.10 ± 0.20	1.43 ± 0.18	0.59 ± 0.06
	25	1.47 ± 0.23	4.46 ± 0.37	2.21 ± 0.21	1.85 ± 0.22	1.53 ± 0.33	0.42 ± 0.07
	50	1.08 ± 0.06	3.28 ± 0.23	1.43 ± 0.27	1.16 ± 0.08	1.32 ± 0.20	0.35 ± 0.01
LSD_0.05_		0.30	0.56	0.38	0.27	0.45	0.09

*Note:* The plant types were IA— *I. aquatica* , LT—*L*. *taraxacifoliar*, PV— *P. vaginatum* , SP— *S. portulacastrum,* and TT— *T. triangulare* . The salt concentrations applied were 0, 25, and 50 dS/m. The values represent the means of four replicates with standard deviations (SD).

An increase in salt concentration reduced the SDW of *I. aquatica* , *L. taraxacifolia*, and *T. triangulare* by 2.16, 1.35, and 1.4 g in UCC soil and by 0.63, 0.66, and 1.4 g in sea sand, respectively. Similarly, increasing salt concentration reduced the RDW of these plants by 0.63, 1.05, and 1.6 g in UCC soil and by 0.60, 0.49, and 0.92 g in sea sand, respectively. However, increased salt concentrations increased the SDW of *S. portulacastrum* and *P. vaginatum* by 7.09 and 4.01 g in UCC soil and by 2.12 and 2.04 g in sea sand, respectively. Similarly, the RDW of *P. vaginatum* was increased by 1.39 g in UCC soil and by 1.17 g in sea sand (Table 3).

Similarly, the root‐to‐shoot ratio (R/S) was significantly affected by the soil and salt concentrations and differed among the plant types (*p* < 0.001). However, the interactions of these three factors had no significant effect on the R:S ratio (*p* = 0.06). *Lactuca taraxacifolia* and *T. triangulare* had the highest R/S ratio (1.05 and 0.94, respectively), while *S. portulacastrum* had the lowest (0.1). Sea sand recorded a 193‐fold increase in R/S ratio over UCC soil. Generally, higher salt concentrations led to a lower R/S ratio in all plant types (0.11‐fold reduction) except for *P. vaginatum* , which recorded a 0.04‐fold higher R/S ratio in UCC soil and a 0.06‐fold higher R/S ratio in sea sand (Table 3).

### 3.3 Tissue Water Content (TWC)

**FIGURE 4:**
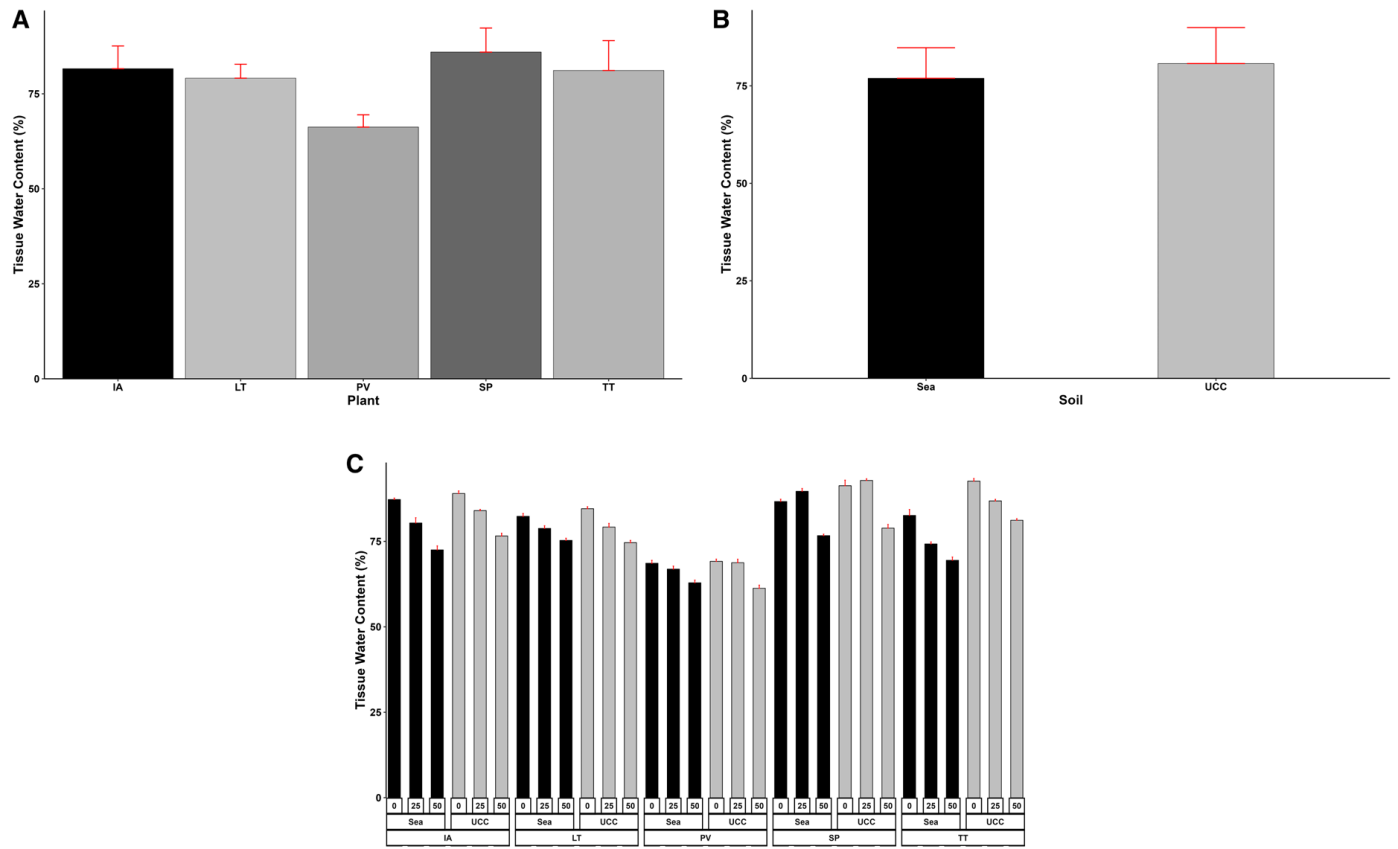
Tissue water content (%) of (A) Plant types (IA—*I. aquatica*, LT—*L*. *taraxacifoliar*, PV— *P. vaginatum* , SP— *S. portulacastrum* , and TT— *T. triangulare* ), (B) Soil Types (UCC, Sea), and (C) Combined plant and soil types under salt concentrations (0, 25, 50 dS/m). Values represent means ± SD.

Tissue water content of the various plant types was significantly affected (*p* < 0.001) by both salt concentrations and soil types. The plant types also exhibited significantly high (*p* < 0.001) differences in the amount of water in their tissues, and the interactive effects of the three main factors were also highly significant (*p* < 0.001). *S. portulacastrum* had the highest TWC, 5%, 6%, 9%, and 30% higher than *I. aquatica* , *T. triangulare* , *L*. *taraxacifoliar*, and *P. vaginatum*, respectively (Figure 4A). For soil types, sea sand reduced the TWC of the plant species by 5% (Figure 4B). Specifically, TWC of *I. aquatica* , *L*. *taraxacifoliar*, *P. vaginatum* , *S. portulacastrum* , and *T. triangulare* were reduced by 4%, 0.8%, 0.4%, 4%, and 15%, respectively, in sea sand. Similarly, salt concentrations generally reduced TWC across all plant types by 4% and 14% at 25 and 50 dS/m concentrations, respectively. However, *S. portulacastrum* recorded a 3% higher TWC at 25 dS/m salt concentration (Figure 4C).

### 3.4 Leaf and Root Sodium (Na) and Chlorine (Cl) Content

**FIGURE 5:**
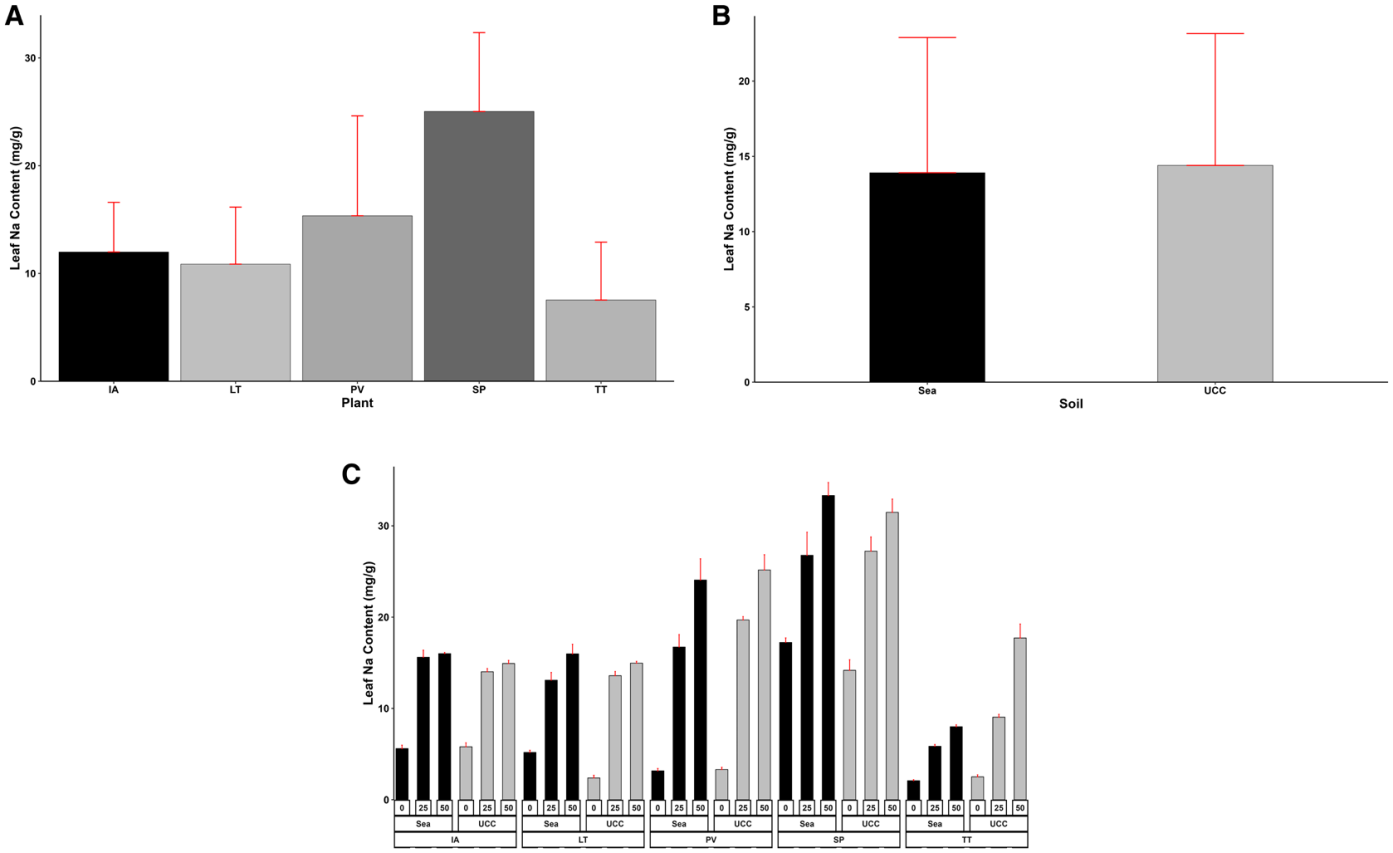
Leaf sodium (Na) content (mg/g) of (A) Plant types (IA— *I. aquatica* , LT—*L*. *taraxacifolia*, PV— *P. vaginatum* , SP— *S. portulacastrum* , and TT— *T. triangulare* ), (B) Soil types (UCC, Sea), and (C) Combined plant and soil types under Salt Concentrations (0, 25, 50 dS/m). Values represent means ± SD.

The plant type (*p* < 0.001), soil type (*p* < 0.05 for Na and *p* < 0.01 for Cl), and salt concentration (*p* < 0.001) significantly affected the Na and Cl contents of the plant leaves. The interactions among these factors were also highly significant (*p* < 0.001). *Sesuvium portulacastrum* (25.03 mg/g) recorded the highest leaf Na content, which was 0.63, 1.09, 1.3, and 2.32‐fold higher compared to *P. vaginatum* , *I. aquatica, L*. *taraxacifoliar*, and *T. triangulare*, respectively (Figure 5A). Leaf Na content increased with increasing salt concentrations. At 25 dS/m salt concentration, *S. portulacastrum* accumulated 0.48, 1.02, 0.82, and 2.62‐fold more leaf Na contents compared to *P. vaginatum* , *I. aquatica, L*. *taraxacifoliar*, and *T. triangulare*, respectively, and 0.32, 1.09, 1.1, and 3.3‐fold more at 50 dS/m salt concentration. On average, UCC soil resulted in a 0.04‐fold increase in leaf Na content compared to sea sand (Figure 5B). However, *L. taraxacifoliar* (1.17 and 0.07‐fold higher at 0 and 50 dS/m salt concentrations, respectively) and *S. portulacastrum* (0.22 and 0.06‐fold higher at 0 and 50 dS/m salt concentrations, respectively) in sea sand recorded higher leaf Na contents at 0 and 50 dS/m salt concentrations (Figure 5C).

**FIGURE 6:**
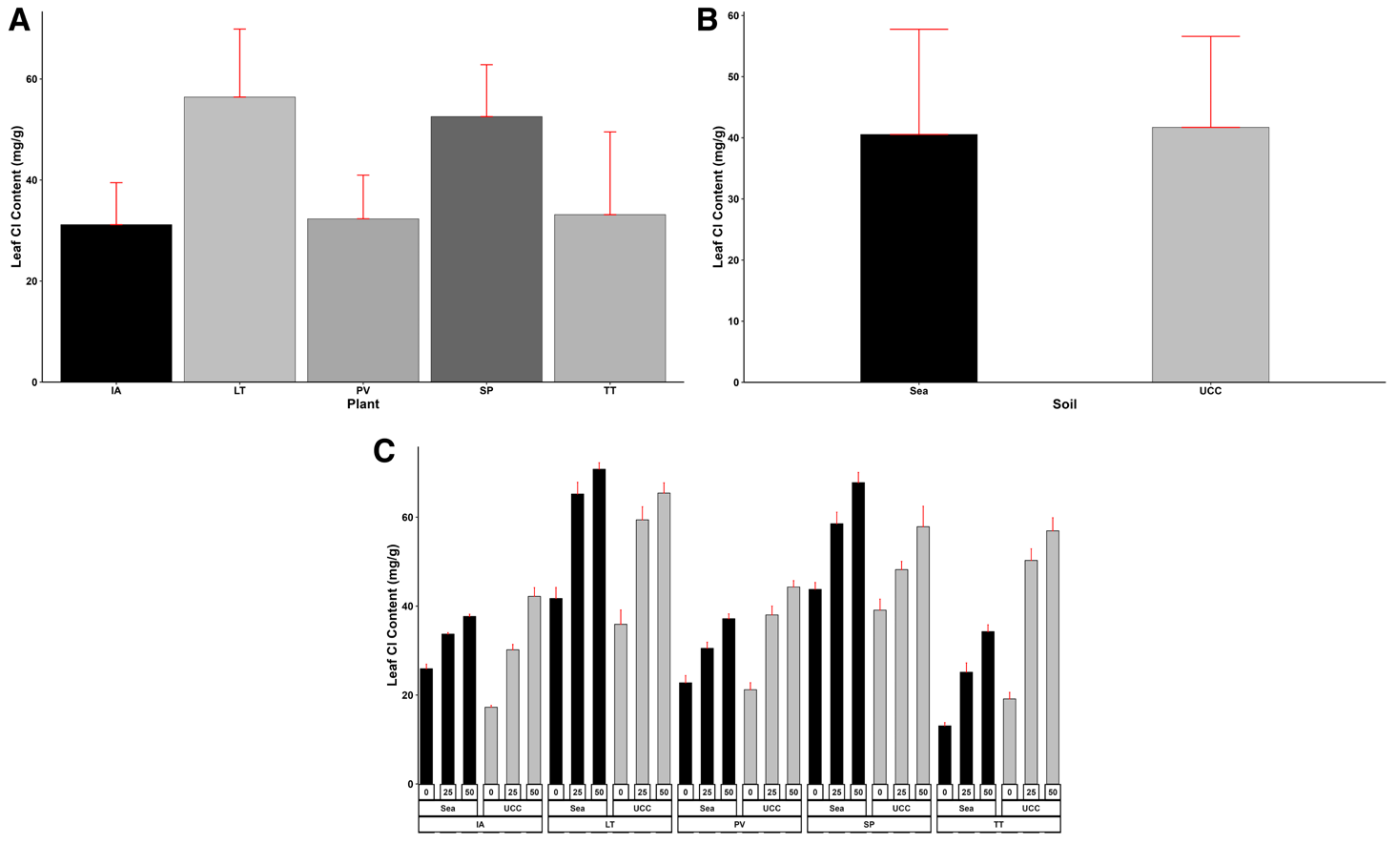
Leaf chlorine (Cl) content (mg/g) of (A) Plant types (IA— *I. aquatica* , LT—*L*. *taraxacifoliar*, PV— *P. vaginatum* , SP— *S. portulacastrum,* and TT— *T. triangulare* ), (B) Soil types (UCC, Sea), and (C) Combined plant and soil types under salt concentrations (0, 25, 50 dS/m). Values represent means ± SD.

Similarly, *L*. *taraxacifoliar* accumulated the most leaf Cl content and was 0.07, 0.7, 0.74, and 0.81‐fold more than *S. portulacastrum* , *T. triangulare* , *P. vaginatum* , and *I. aquatica*, respectively (Figure 6A). UCC soil increased leaf Cl content, 0.03‐fold higher than sea sand (Figure 6B). At 25 dS/m salt concentration, *L. taraxacifoliar* was found to have accumulated more Cl content compared to *S. portulacastrum* (0.17‐fold more), *T. triangulare* (0.65‐fold more), *P. vaginatum* (0.82‐fold more), and *I. aquatica* (0.95‐fold more). Likewise, at 50 dS/m treatment, it accumulated 0.08, 0.49, 0.67, and 0.71‐fold higher Cl contents, respectively (Figure 6C).

**FIGURE 7:**
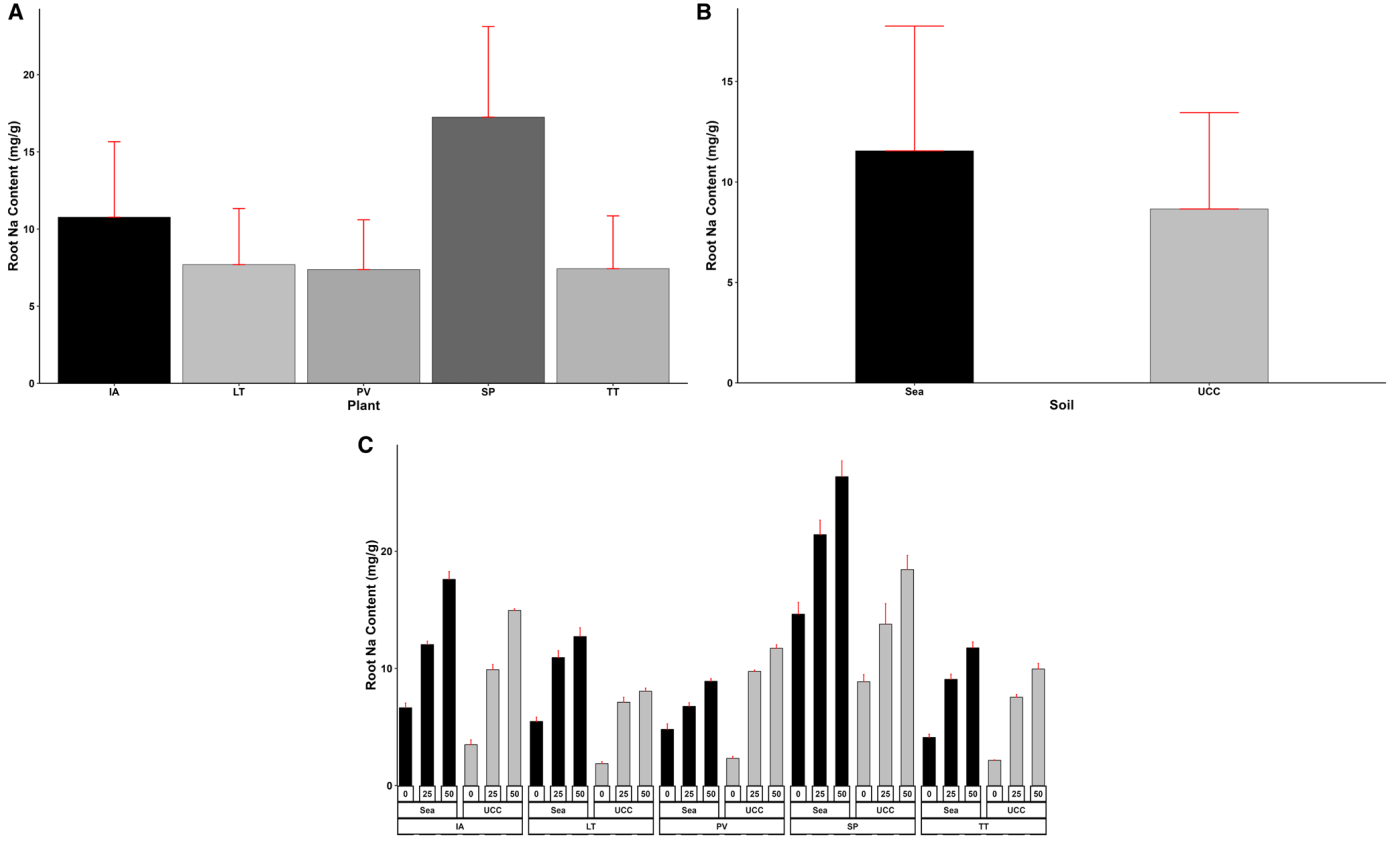
Root sodium (Na) Content (mg/g) of (A) Plant types (IA—*I. aquatica*, LT—*L*. *taraxacifoliar*, PV— *P. vaginatum* , SP— *S. portulacastrum,* and TT— *T. triangulare* ), (B) Soil types (UCC, Sea), and (C) Combined plant and soil types under salt concentrations (0, 25, 50 dS/m). Values represent means ± SD.

Root Na and Cl content varied significantly among different plant types, soil types, and salt concentrations and their various interactions (*p* < 0.001). Plants in sea sand (11.55 mg/g) were observed to have accumulated more Na ions in their roots than in UCC soil (8.66 mg/g) (Figure 7B). Generally, the increase in salt concentrations led to significant increases in both root Na and Cl contents across all plant types. *Sesuvum portulacastrum* (17.25 mg/g) accumulated the highest root Na content compared to *I. aquatica* (10.77 mg/g), *L. taraxacifoliar* (7.69 mg/g), *T. triangulare* (7.43 mg/g), and *P. vaginatum* (7.38 mg/g) (Figure 7A). At 25 dS/m salt concentration, *S. portulacastrum* accumulated 61%, 95%, 112%, and 113% more Na in the root compared to *I. aquatica* , *L*. *taraxacifoliar*, *T. triangulare* , and *P. vaginatum*, respectively. Similarly, at 50 dS/m salt concentration, the quantity of Na in *S. portulacastrum* roots was found to be 38%, 116%, 106%, and 117% higher than the Na of *I. aquatica* , *L*. *taraxacifoliar*, *T. triangulare* , and *P. vaginatum*, respectively (Figure 7C).

**FIGURE 8:**
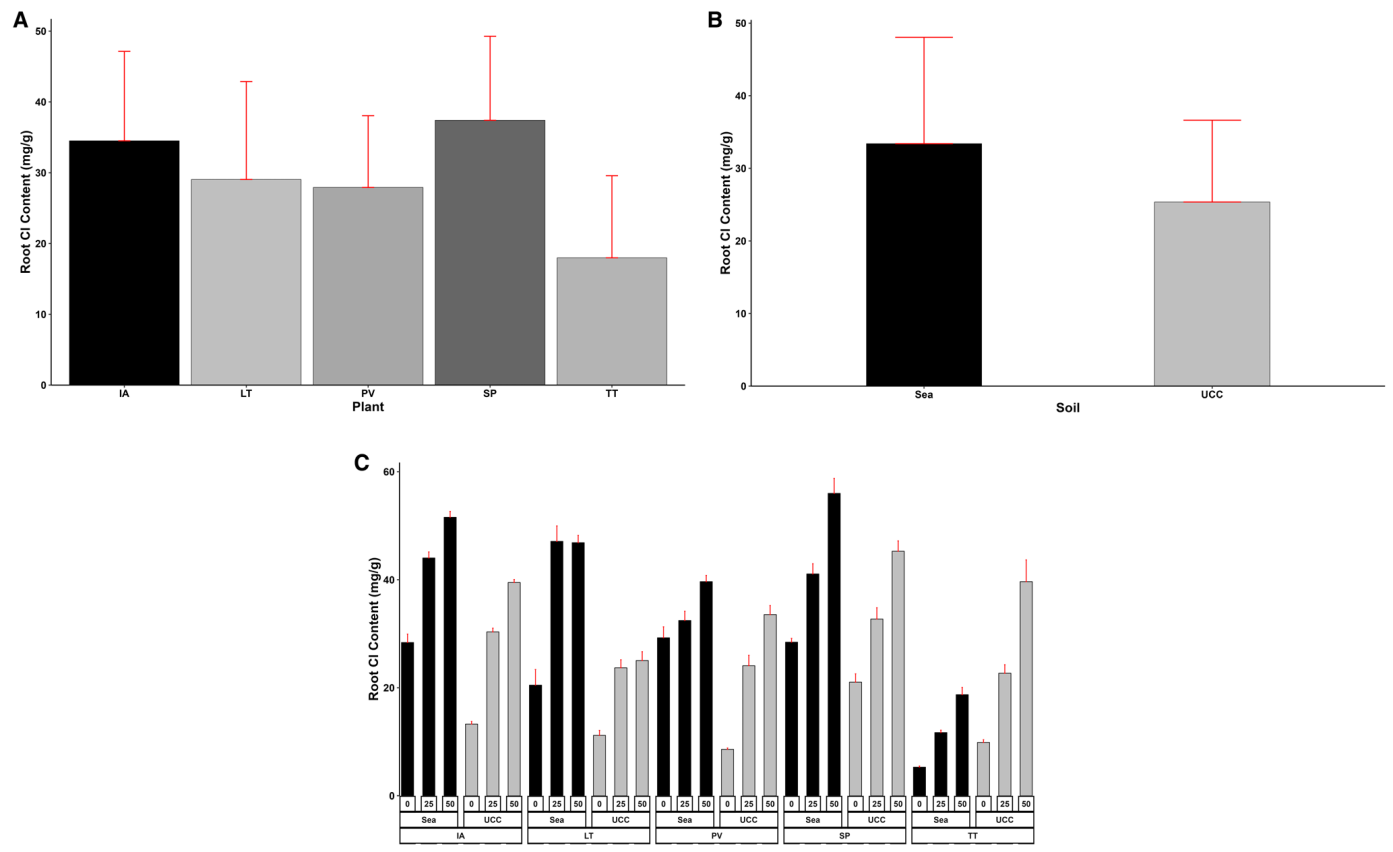
Root chlorine (Cl) content (mg/g) of (A) Plant types (IA—*I. aquatica*, LT—*L*. *taraxacifoliar*, PV— *P. vaginatum* , SP— *S. portulacastrum* , and TT— *T. triangulare* ), (B) Soil types (UCC, Sea), and (C) Combined plant and soil types under salt concentrations (0, 25, 50 dS/m). Values represent means ± SD.

Meanwhile, for root Cl content also, *S. portulacastrum* (37.42 mg/g) accumulated 8%, 108%, 29%, and 34% higher contents compared to *I. aquatica* , *T. triangulare, L*. *taraxacifoliar*, and *P. vaginatum*, respectively (Figure 8A). Regarding soil types, plants in sea sand were shown to have accumulated 32% more Cl in their root compared to UCC soil (Figure 8B). Interestingly, *I. aquatica* recorded the highest Cl accumulation in the root at 25 dS/m salt concentration, followed by *S. portulacastrum* (1% lower), *L*. *taraxacifoliar* (1% lower), *P. vaginatum* (32% lower), and *T. triangulare* (116% lower). At 50 dS/m treatment, *S. portulacastrum* recorded the highest root Cl content and was found to be 11%, 73%, 41%, and 38% more than *I. aquatica* , *T. triangulare, L*. *taraxacifoliar*, and *P. vaginatum*, respectively (Figure 8C).

### 3.5 Leaf and Root Sodium (Na) and Chlorine (Cl) Uptake

**FIGURE 9:**
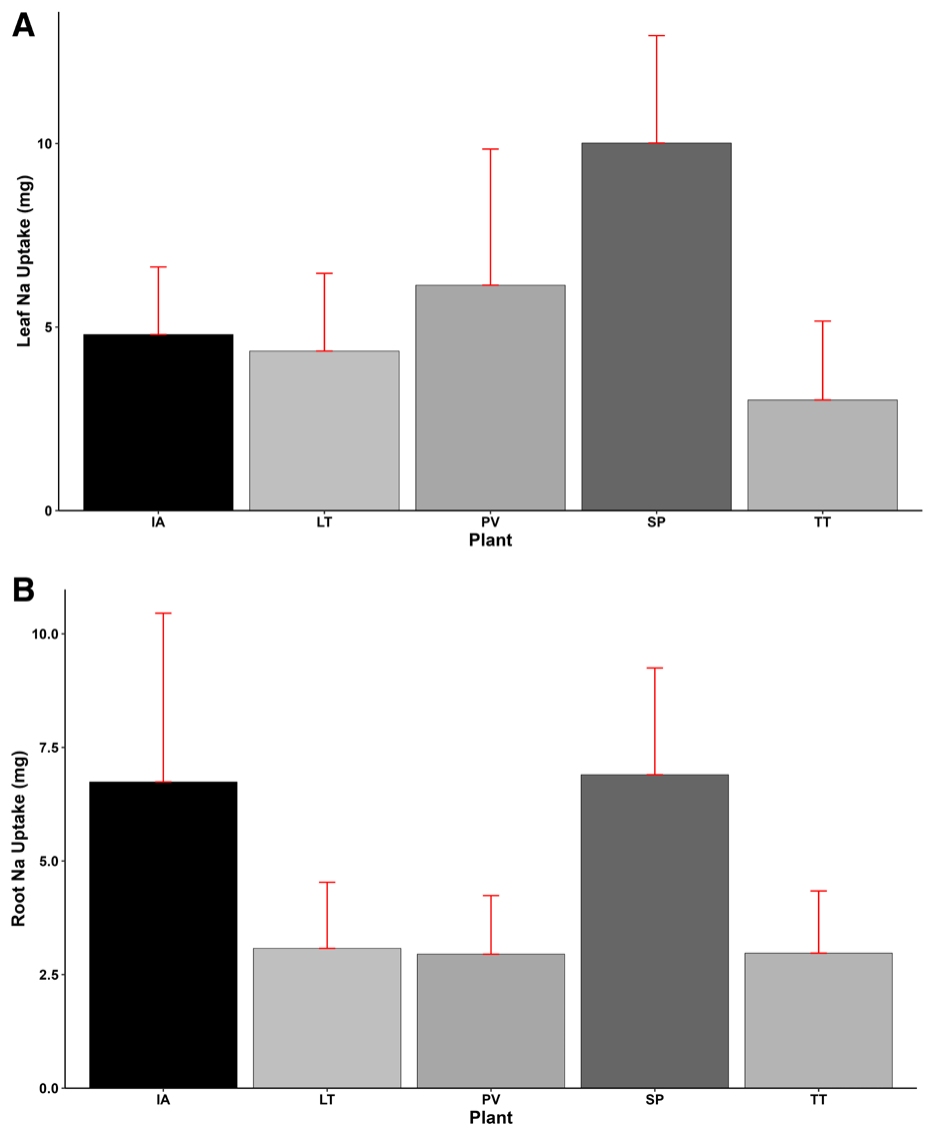
(A) Leaf Na uptake, (B) Root Na uptake of plant types (IA— *I. aquatica* , LT—*L*. *taraxacifoliar*, PV— *P. vaginatum* , SP— *S. portulacastrum* , and TT— *T. triangulare* ).

Significant variations (*p* < 0.001) in leaf sodium (Na) uptake were observed among the plant species. The highest leaf Na uptake was obtained by *S. portulacastrum* (10.01 mg), followed by *P. vaginatum* (6.14 mg), with *T. triangulare* recording the lowest uptake of 3.01 mg (Figure 9A). Similarly, root Na uptake also differed significantly (*p* < 0.001) across the plant species. Recording the highest root Na uptake of 6.9 mg, *S. portulacastrum* obtained 2%, 124%, 132%, and 134% more uptake than *I. aquatica* , *L*. *taraxacifoliar*, *T. triangulare* , and *P. vaginatum*, respectively. Generally, Na uptake in leaves was higher than in roots for most species, except for *I. aquatica* , where Na was 40% higher in roots than in leaves (Figure 9B).

**FIGURE 10:**
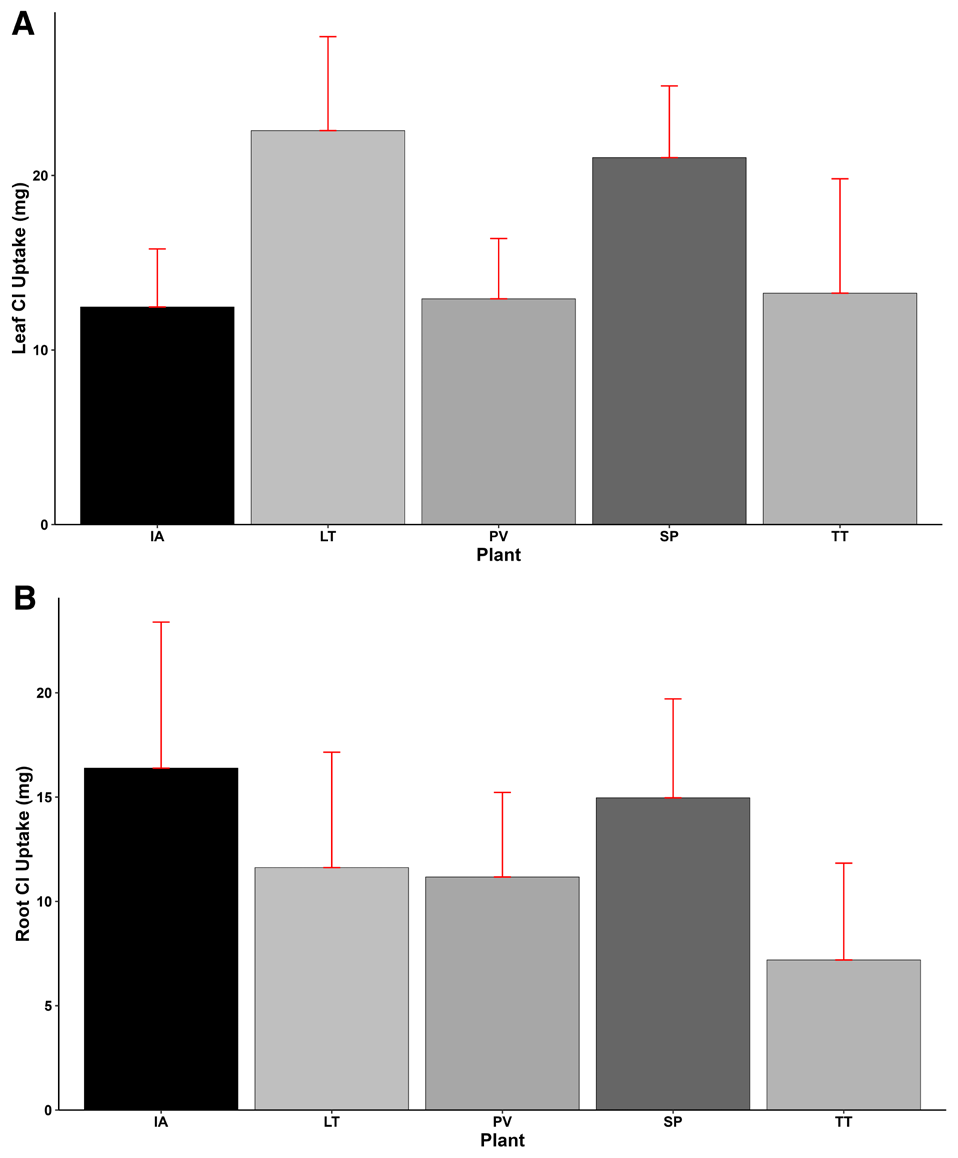
(A) Leaf Cl uptake, (B) Root Cl uptake of plant types (IA— *I. aquatica* , LT—*L*. *taraxacifoliar*, PV— *P. vaginatum* , SP— *S. portulacastrum* , and TT— *T. triangulare* ).

Leaf Cl uptake showed significant differences (*p* < 0.001) among the plant species, with *L*. *taraxacifoliar* (22.57 mg) showing the highest Cl uptake, followed by *S. portulacastrum* (21.02 mg), *T. triangulare* (13.26 mg), *P. vaginatum* (12.93 mg), and *I. aquatica* (12.47 mg), having the lowest leaf Cl uptake (Figure 10A). Similarly, significant variations (*p* < 0.001) were found in root Cl content among the plant species. The highest and lowest root Cl uptake of 16.39 and 7.19 mg were obtained by *I. aquatica* and *T. triangulare* , respectively. *Sesuvium portulacastrum* , having a root Cl uptake of 14.97, was 9% lower than *I. aquatica* and 29%, 34%, and 108% higher than *L*. *taraxacifoliar*, *P. vaginatum* , and *T. triangulare*, respectively. Similar to Na uptake, Cl uptake in leaves was generally higher than that of roots, except for *I. aquatica* , which showed a contrary trend of 31% more uptake in roots (Figure 10B).

### 3.6 Soil Electrical Conductivity, pH, Sodium (Na) Content, and Chlorine (Cl) Content

**FIGURE 11:**
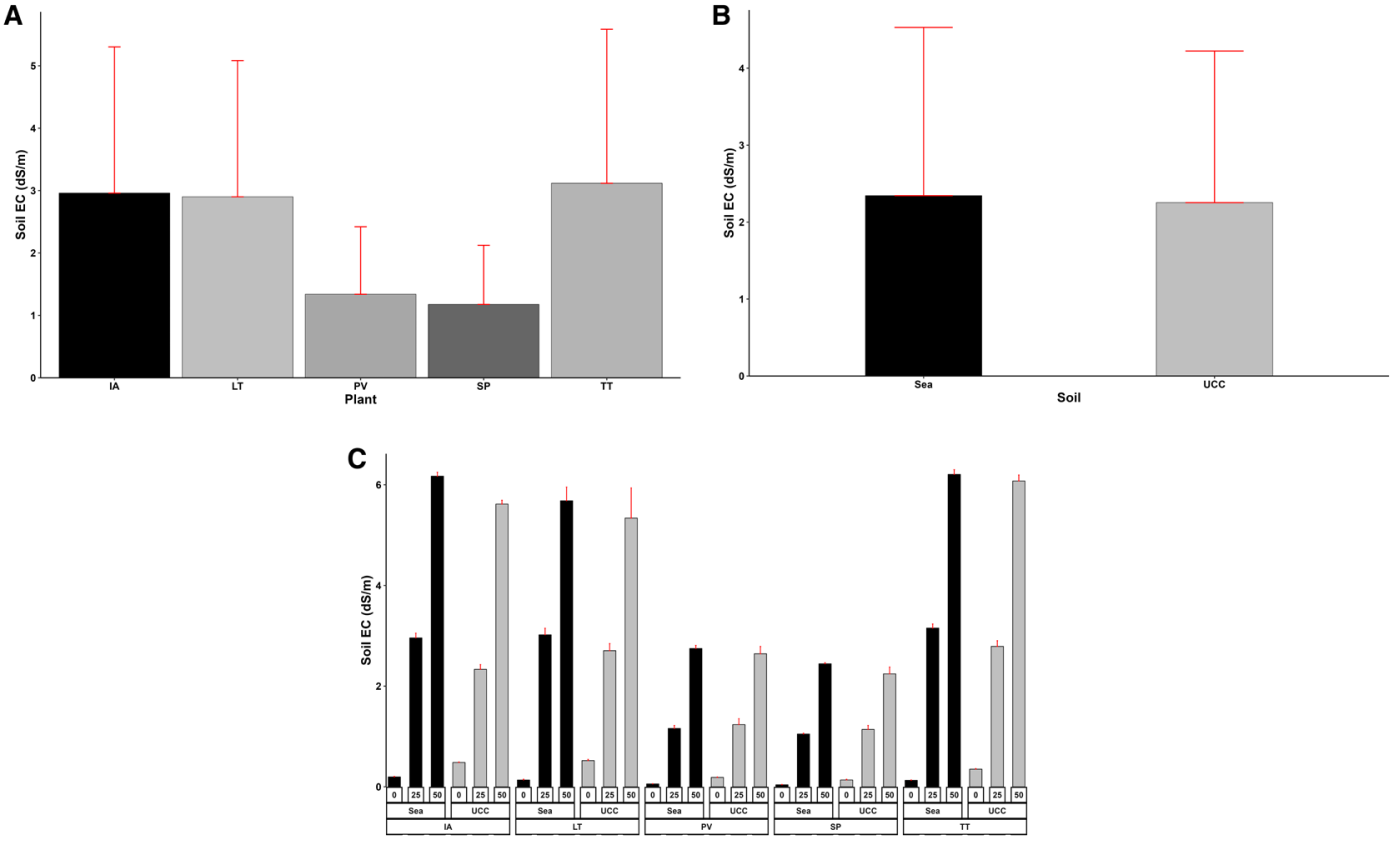
Soil electrical conductivity (dS/m) of (A) Plant types (IA— *I. aquatica* , LT—*L*. *taraxacifoliar*, PV— *P. vaginatum* , SP— *S. portulacastrum* , and TT— *T. triangulare* ), (B) Soil types (UCC, Sea), and (C) Combined plant and soil types under salt concentrations (0, 25, 50 dS/m). Values represent means ± SD.

Soil EC was significantly affected by the treatments and their interactions (*p* < 0.001). The effects of the salt concentration on soil EC were very obvious, as no salt concentration (i.e., 0 dS/m) had EC values 9.4‐fold and 20‐fold lower compared to 25 and 50 dS/m concentrations, respectively. Sea sand generally had EC values 4% higher than UCC soil (Figure 11B). In terms of the plant species, *S. portulacastrum* resulted in the lowest soil EC across all the plant types, 14%, 145%, 151%, and 164% lower compared to *P. vaginatum* , *L*. *taraxacifoliar*, *I. aquatica* , and *T. triangulare*, respectively (Figure 11A). The ECs of soils that contained *I. aquatica* , *L*. *taraxacifoliar*, and *T. triangulare* at 50 dS/m salt concentration were observed to be very high in both sea sand (6.17, 5.68, and 6.21 dS/m, respectively) and UCC soils (5.62, 5.34, and 6.08 dS/m, respectively), whereas those of *P. vaginatum* and *S. portulacastrum* at 50 dS/m salt concentrations recorded ECs of 2.75 and 2.44 dS/m in sea sand and 2.64 and 2.24 dS/m in UCC soils (Figure 11C).

**FIGURE 12:**
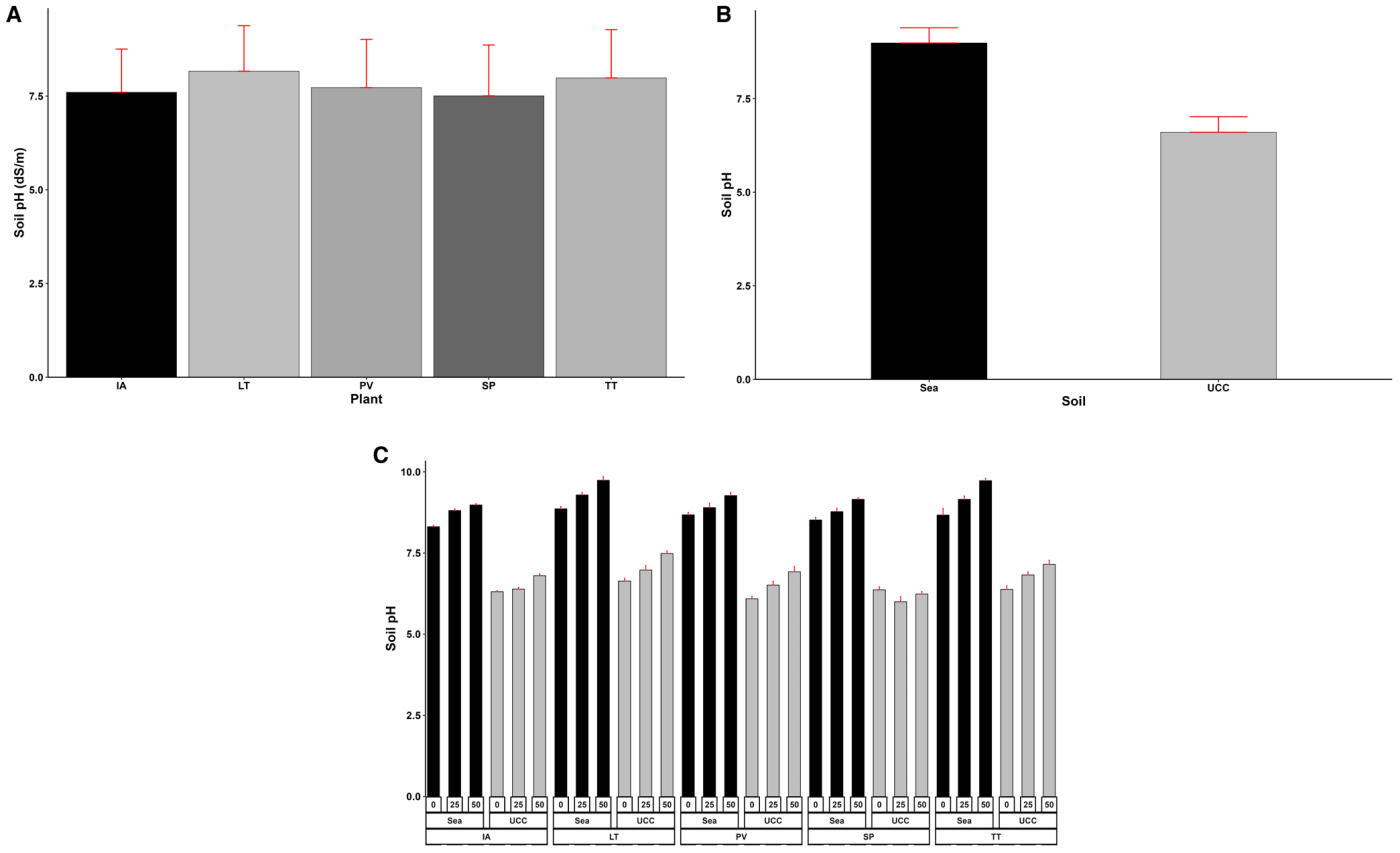
Soil pH of (A) Plant types (IA— *I. aquatica* , LT—*L*. *taraxacifoliar*, PV— *P. vaginatum* , SP— *S. portulacastrum* , and TT— *T. triangulare* ), (B) Soil types (UCC, Sea), and (C) Combined plant and soil types under salt concentrations (0, 25, 50 dS/m). Values represent means ± SD.

Similarly, soil pH was significantly affected by all the treatments and their interactions (*p* < 0.001). Generally, sea sand (9) had significantly higher (36% higher) soil pH than UCC soil (6.6) (Figure 12A). Soil pH recorded 4% and 9% higher values at increasing salt concentrations of 25 dS/m (7.76) and 50 dS/m (8.15), respectively (Figure 12C). *Sesuvium portulacastrum* succeeded in reducing soil pH to an average of 7.51, which was 1%, 3%, 6%, and 9% lower compared to *I. aquatica* (7.6), *P. vaginatum* (7.73), *T. triangulare* (7.98), and *L*. *taraxacifoliar* (8.16), respectively (Figure 12B).

**FIGURE 13:**
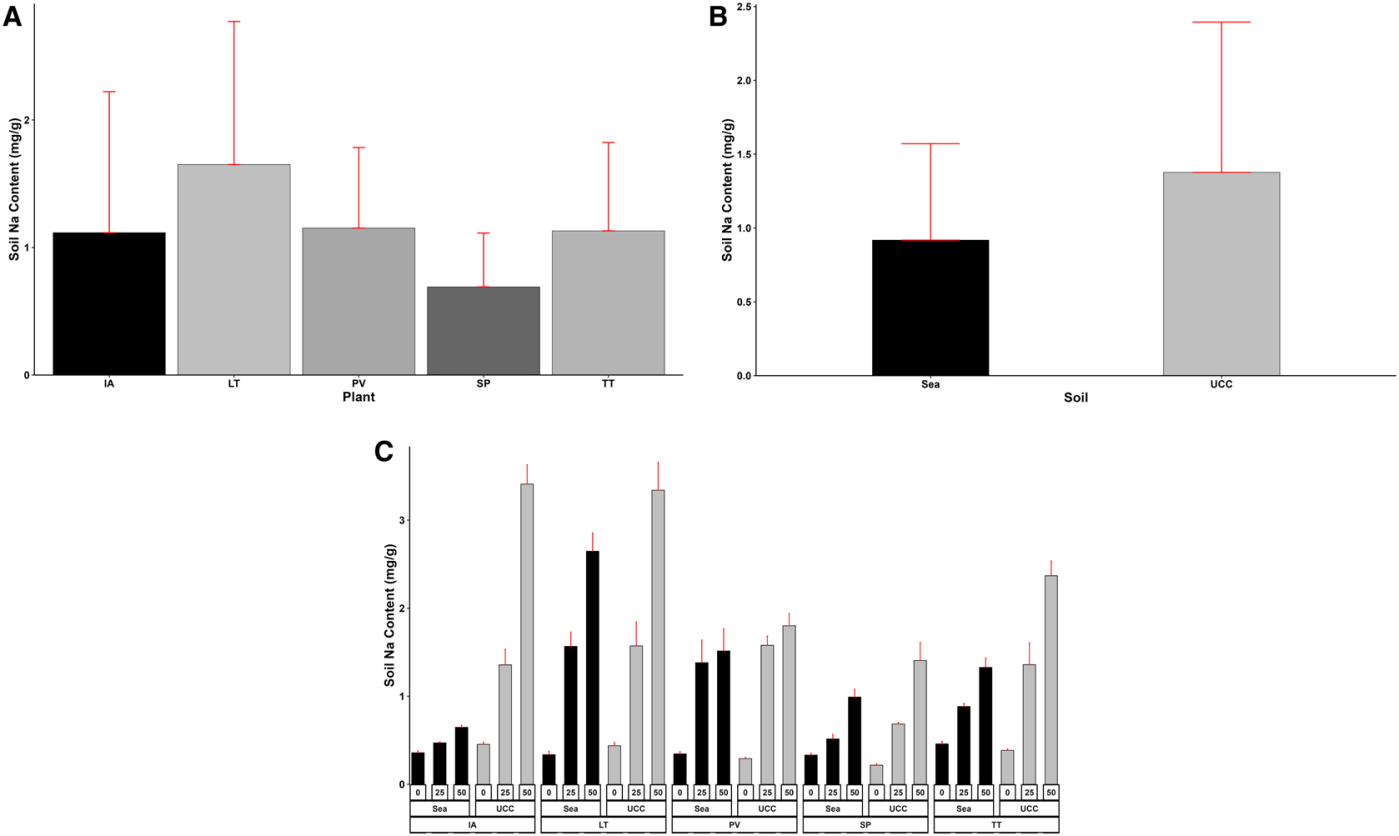
Soil Sodium (Na) Content (mg/g) of (A) Plant Types (IA—*I. aquatica*, LT—*L*. *taraxacifoliar*, PV— *P. vaginatum* , SP— *S. portulacastrum* , and TT— *T. triangulare* ), (B) Soil types (UCC, Sea), and (C) Combined plant and soil types under Salt Concentrations (0, 25, 50 dS/m). Values represent means ± SD.

The treatment factors and interactions had a significant effect on the final soil Na and Cl contents (*p* < 0.001). Sea sand accumulated a Na content of 0.92 mg/g, 50% lower than the Na content in UCC soil (1.38 mg/g) (Figure 13B). Additionally, increasing salt concentrations increased both the soil's Na and Cl contents. The 0 dS/m salt concentration (0.36 mg/g) resulted in soil Na content values 217% and 442% lower than Na contents at 25 dS/m (1.14 mg/g) and 50 dS/m (1.95 mg/g) salt concentrations, respectively. In addition, *S. portulacastrum* , recording a mean Na content of 0.69 mg/g, was found to be 62%, 64%, 67%, and 139% lower compared to *I. aquatica* , *T. triangulare* , *P. vaginatum* , and *L. taraxacifoliar*, respectively (Figure 13A). Even at high salt concentrations, *P. vaginatum* and *S. portulacastrum* significantly lowered soil Na contents. At 25 and 50 dS/m salt concentrations, *P. vaginatum* recorded Na contents of 1.38 and 1.52 mg/g in sea sand and 1.58 and 1.8 mg/g in UCC soil. Similarly, in *S. portulacastrum* , soil Na contents stood at 0.52 and 0.99 mg/g in sea sand and 0.69 and 1.41 mg/g in UCC soil at 25 and 50 dS/m salt concentrations, respectively (Figure 13C).

**FIGURE 14:**
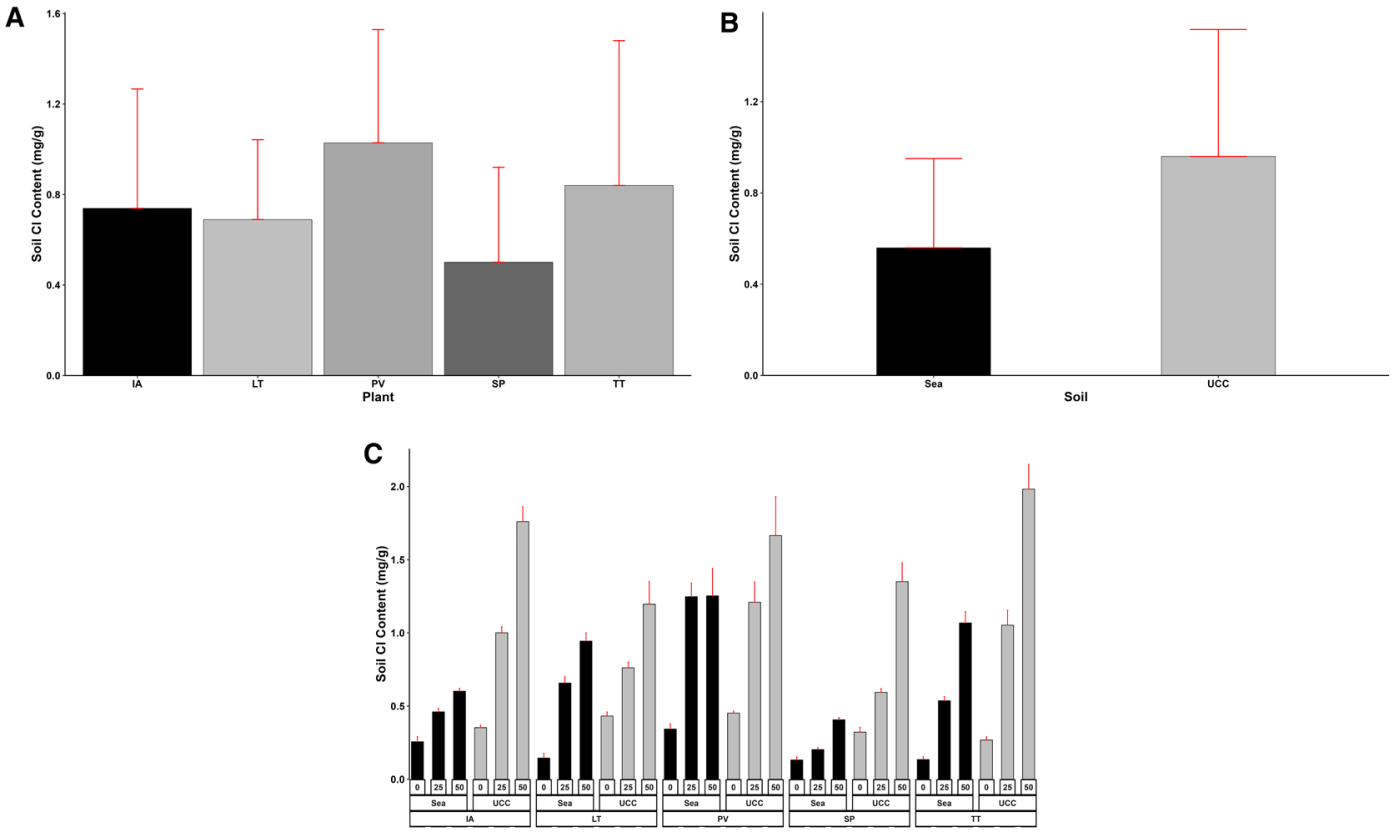
Soil chlorine (Cl) content (mg/g) of (A) Plant types (IA— *I. aquatica* , LT—*L*. *taraxacifoliar*, PV— *P. vaginatum* , SP— *S. portulacastrum* , and TT— *T. triangulare* ), (B) Soil types (UCC, Sea), and (C) Combined plant and soil types under salt concentrations (0, 25, 50 dS/m). Values represent means ± SD.

Like soil Na content, soil Cl content was 0.71‐fold higher in UCC soil (0.96 mg/g) than in sea sand (0.56 mg/g) (Figure 14B). In addition, soil Cl content at 0 dS/m salt concentration was 1.75 and 3.36‐fold lower than Cl contents at 25 and 50 dS/m salt concentrations, respectively. Regarding plant types, *S. portulacastrum* accumulated the least soil Cl content of 0.5 mg/g, which was 0.38, 0.48, 0.68, and 1.06‐fold lower than that of *L*. *taraxacifoliar*, *I. aquatica, T. triangulare*, and *P. vaginatum*, respectively (Figure 14A). While *S. portulacastrum* in sea sand at 0 dS/m salt concentration recorded the lowest soil Cl content of 0.13 mg/g, *T. triangulare* in UCC soil at 50 dS/m salt concentration accumulated the highest soil Cl content (1.98 mg/g), 13.85‐fold more (Figure 14C).

## 4 Discussion

### 4.1 Halophyte Diversity and Adaptability

The study showed different groups of salt‐tolerant species with varying growth habits, life cycles, and potential applications. The diverse growth habits and life cycles of the identified halophytes, including herbaceous plants, shrubs, and succulents, demonstrate the varied strategies these plants have evolved to thrive in saline conditions. This diversity suggests that different species may be suitable for various applications in soil remediation and sustainable agriculture, depending on specific environmental conditions and management goals. Thus, the various herbaceous plants, shrubs, succulents, climbers, and trees highlight the variety of plant life in Ghana's saline habitats. The predominance of herbaceous plants and upright growth habits suggests that these forms may offer particular advantages, such as competition for resources and enhanced soil stability in saline environments. The presence of the identified halophytes is crucial for the overall resilience and ecological stability of these areas.

Among the halophytes identified in this study, *Sesuvium portulacastrum* , *Paspalum vaginatum* , *Indigofera spicata,* and *Cyperus ligularis* share similarities with those documented by Swaine et al. (1979) who studied the zonation of halophytes along the coast in Ghana. It is essential to note that Swaine et al.'s research focused on the coastal region west of Accra, specifically Bortiano, whereas the current research was conducted in Cape Coast. This signifies the resilience and adaptability of halophytic species across diverse coastal environments and periods, as indicated by Wungrampha et al. (2021). The halophytes identified belong to various families and exhibit a range of growth habits, from herbaceous plants like *Amaranth* spp. and *Cyperus ligularis* to shrubs like *Capraria biflora* and succulents like *Sesuvium portulacastrum* and *Sansevieria masoniana*. Also, in terms of lifecycles, annuals such as *Ipomoea asarifolia* , *Lactuca taraxacifolia*, and perennials, including *Paspalum vaginatum* and *Cocos nucifera,* were identified. These growth habits and life cycles reflect halophytes' various ecological strategies for adapting to their environments. Likewise, Mehra et al. (2024) observed varied growth habits and lifecycles in halophytes and noted their role in conferring salt stress tolerance in halophytes.

The different growth habits reflect the evolutionary strategies of these plants, providing opportunities for various applications. The differences in growth habits and lifecycles observed in the identified halophytes, for example, contribute to the ecological resilience of coastal ecosystems. Plants like *Opuntia* spp. (Ciriminna et al. 2019), *Ipomoea asarifolia* (Albuquerque et al. 2007), *Sesuvium portulacastrum* (He et al. 2022), *Cyperus ligularis* (Casierra‐Martínez et al. 2017), and *Canavalia rosea* (Lin et al. 2021) play crucial roles in ground cover, erosion control, and soil stabilization, while others, including *Ipomoea aquatica* (Rai and Sinha 2001), *Pedalium murex* (Tripathi et al. 2021), and *Sesuvium portulacastrum* (He et al. 2022) are valuable for phytoremediation and restoration of degraded soils. Additionally, the adaptation of certain species, such as *Paspalum vaginatum* (Fabbri et al. 2016) for turfgrass and forage for livestock, and *Indigofera spicata* (Mouafon et al. 2021) for soil improvement and forage for livestock, highlights the diverse potential these halophytes possess in sustainable land management and ecological restoration.

The identified halophytes hold significant economic, ecological, and cultural importance. Several species, such as *Amaranth* spp. (Aderibigbe et al. 2022), *Capraria biflora* (Vasconcellos et al. 2005) and *Euphorbia albomarginata* (Rojas 2010) have documented uses in traditional medicine, while others serve culinary purposes, like the edible leaves of *Sesuvium portulacastrum* (Lokhande et al. 2013) and *Talinum triangulare* (Pavithra et al. 2017), as well as the fruits of *Physalis* spp. (Daunay et al. 2006) and *Cocos nucifera* (Pham 2016). Ornamental value is evident in plants like *Sansevieria masoniana* (Chahinian 2000; Rêgo et al. 2020) and *Kalanchoe daigremontiana* (Navarrete et al. 2021). Conserving and applying these plant species is essential for sustainable land use, biodiversity conservation efforts, and contributing to Ghana's local economies. Further research and conservation efforts should aim to preserve and exploit this rich diversity of halophytic plant types and their various qualities.

### 4.2 Effects of Soil Type on Growth of Selected Halophytes

The study revealed significant variations in growth parameters and salt removal efficiency among the tested halophytes, with *P. vaginatum* and *S. portulacastrum* demonstrating superior performance across multiple metrics. These findings align with their known salt tolerance mechanisms and potential for phytoremediation.

Soil type significantly affected most of the measured parameters, with UCC soil, an arable soil, generally promoting better halophyte growth than sea sand. However, general knowledge suggests that halophytes thrive in sea sand due to their inherent salt tolerance. The superior performance of UCC soil may be attributed to its improved structure, better drainage, and higher nutrient availability compared to sea sand, which is poor in structure and nutrient composition. Morgan and Connolly (2013) highlighted that variations in soil properties, such as texture, organic matter content, and nutrient levels, can profoundly impact plant performance and productivity. Arable soil may have provided halophytes with a more balanced environment, enabling optimal physiological functions and biomass production.

Interestingly, arable soil resulted in a significantly lower R/S ratio than the sea sand, suggesting that these plants allocated fewer resources to root development under favorable conditions. In contrast, the poor nutrient composition of the sand likely compelled plants to allocate more resources to the roots to scavenge for water and nutrients. This aligns with resource allocation theories and the findings of Wang et al. (2023) and Hermans et al. (2006), which emphasizes increased root biomass allocation in resource‐limited environments. Nevertheless, the lower performance in sea sand raises questions about whether these species prioritize salinity tolerance over nutrient acquisition. It is also probable that the specific species under study were not well adapted to the extreme nutrient limitations of sea sand or that the experimental conditions did not fully mimic natural saline habitats where halophytes thrive. Future research could investigate whether amending sea sand with organic matter or nutrients enhances the performance of halophytes.

The results revealed significant salt concentrations and soil type effects on all plant species' shoot dry weight (SDW) and root dry weight (RDW). As expected, the increase in salt concentration generally led to a reduction in both SDW and RDW in *T. triangulare* , *I. aquatica* , and *L. taraxacifolia*, suggesting osmotic stress and ion toxicity (Amirjani 2011; Garg et al. 2006; De Pascale et al. 2003). However, *P. vaginatum* and *S. portulacastrum* exhibited increased SDW at higher salt concentrations, challenging the notion that salt stress generally limits plant biomass accumulation. This unique response suggests species‐specific adaptations, such as exploiting higher salinity for growth, as reported by Lokhande et al. (2013). In their study, *S. portulacastrum* demonstrated increased biomass accumulation under salinity conditions between 100 and 400 mM NaCl and nutrient‐deprived settings, indicating the plant's capacity to adapt to and benefit from elevated salinity levels. This highlights the role of halophytic adaptations such as selective ion uptake, osmoregulation, and efficient resource allocation in maintaining growth under saline environments. *Paspalum vaginatum's* demonstration of increased RDW under high salt concentrations suggests a compensatory strategy for sustaining water uptake despite elevated soil osmolarity. Such responses are indicative of species‐specific physiological adaptations, such as selective ion uptake, ion compartmentalization, and salt‐stimulated metabolic activity (Slama et al. 2006, 2007, 2008; Yildirim and Güvenç 2006; Shahba et al. 2012). The discrepancies observed in the biomass responses of different plant species to salt stress may be attributed to various factors, including the frequency and duration of salt concentration applications. Unlike previous studies involving periodic salt concentrations, the salt concentration in this experiment was applied only once, potentially influencing the plant's physiological responses. These results warrant further investigation to confirm whether biomass increases correspond to enhanced ion sequestration and water capacity.

The root‐to‐shoot (R/S) ratio was also significantly influenced by soil types and salt concentrations, indicating the plasticity of halophyte root–shoot allocation strategies in response to environmental conditions. *Talinum triangulare* and *L. taraxacifolia* displayed very high R/S ratios, suggesting a preference for allocating biomass towards root development. This could reflect their adaptation to nutrient or water limitations, as greater root biomass enhances resource acquisition and utilization. In contrast, *S. portulacastrum* had the lowest R/S ratio, indicating the allocation of more biomass to shoots to optimize photosynthetic gain. This could also be attributed to the fact that *T. triangulare* and *L. taraxacifolia* possess taproot systems, which are inherently heavier than the fibrous roots of *S. portulacastrum* . This finding contradicts the finding of Akman (2020), who worked on tap‐rooted plants, including *Pisum sativum* , *Carthamus tinctorius* , and *Vicia pannonica* , and fibrous‐rooted plants, including *Secale cereale* , *Hordeum vulgare* , and *Avena sativa* . They found that the fibrous‐rooted plants have higher root‐to‐shoot ratios than the tap‐rooted plants. These inconsistencies could arise from differences in environmental factors, experimental conditions, or species‐specific adaptations. In this study, the relative nutrient‐poor conditions of sea sand likely compelled *T. triangulare* and *L. taraxacifolia* to allocate more resources to their taproots for survival. Conversely, the low R/S ratio of *S. portulacastrum* might indicate a strategy of maximizing above‐ground growth for photosynthesis and reproduction, especially under more favorable conditions. These findings indicate that biomass allocation in halophytes is not solely dictated by root morphology but by the availability of environmental resources and stress tolerance mechanisms.

The study's results also indicate the significant influence of soil types and salt concentrations on the relative growth rates of the various halophytes. *Paspalum vaginatum* and *S. portulacastrum* demonstrated the highest relative growth rate at increased salt concentrations, suggesting a certain level of tolerance or even potential benefits for these species under elevated salinity conditions. This finding confirms Slama et al.'s (2006, 2007, 2008) work, which reported increased growth rates in *S. portulacastrum* when subjected to low salt concentrations. These findings suggest that optimal salinity conditions may stimulate the physiological processes or metabolic pathways of these species, thereby enhancing growth. However, it contradicts the work of Shahba et al. (2012), whose research indicated a reduced plant height in *Paspalum vaginatum* under salinity stress attributed to increased root mass.

On the contrary, *Talinum triangulare* , *Ipomoea aquatica,* and *Lactuca taraxacifolia* exhibited lower RGR at increasing salt concentrations, indicating lower tolerance to high salt concentrations. This observation suggests that these species may have reached their threshold for salt tolerance at the 25 and 50 dS/m salt concentrations, beyond which their growth is significantly inhibited. It is plausible that the excessively high salt concentrations in the soil surpassed the tolerance mechanisms of these halophytes, leading to compromised growth rates. For instance, excessive accumulation of Na and Cl ions in the rhizosphere can disrupt cellular ion homeostasis, inhibiting enzymes and impairing metabolic processes. These results confirm the findings of Shrivastava and Kumar (2015), Jumberi et al. (2002), Hasanuzzaman et al. (2009) and Sakr et al. (2015), who have reported similar trends in various plants and attributed these changes to ion toxicity, osmotic stress, and nutrient imbalances.

The study's results revealed a consistent trend where tissue water content (TWC) generally decreased with increasing salt concentrations. This aligns with expectations, as higher salinity typically reduces water uptake due to osmotic stress in the rhizosphere (Lu and Fricke 2023). However, *S. portulacastrum* exhibited a contrasting trend, particularly at 25 dS/m salt concentration, where TWC was higher than at 0 and 50 dS/m salt concentrations. This observation suggests that the tolerance of *S. portulacastrum* to salt stress may be attributed to the succulent nature of its leaves, which mitigates osmotic stress. The succulent nature of its leaves allows *S. portulacastrum* to retain and accumulate water in specific vacuoles, potentially enabling it to counteract the effects of increased salt concentrations in its tissues. This finding is consistent with the findings of Vyas et al. (2023) and Khan et al. (2000), who highlighted succulence as a salt tolerance mechanism in other halophytes, including *Suaeda nudiflora*, *Suaeda fruticosa*, and *Salicornia brachiata*. Contrastingly, although *T. triangulare* also possesses succulent leaves, its TWC was reduced at 25 and 50 dS/m salt concentrations. This finding may be attributed to the limited salt tolerance of *T. triangulare* , as the plant has been reported to tolerate salt concentrations lower than 0.5% NaCl as a miohalophyte (Bamidele et al. 2007; Chapman 1942). This finding reveals the variability in salt tolerance mechanisms even among halophytes with common morphological characteristics, suggesting that aside from succulence, other factors, such as osmolyte synthesis and compartmentalization, may play a decisive role in determining species‐specific responses to salinity stress.

The plants' performance index (PI) varied significantly among the salt concentrations. Generally, the PI decreased with increasing salt concentrations, which is consistent with the findings by Giorio and Sellami (2021) and Saddiq et al. (2021) in rice and wheat plants under salt stress. The reduction in PI reflects compromised photosynthetic performance and overall plant health in response to salinity stress. Salt stress may have impaired energy conversion efficiency due to disruptions in electron transport in photosystem II. However, it is noteworthy that *S. portulacastrum* and *P. vaginatum* had increased PI at 25 dS/m salt concentration, indicating a potential enhancement in photosynthetic efficiency under moderate salt stress conditions, which may be attributed to adaptive mechanisms such as enhanced osmotic adjustment and chlorophyll synthesis.

### 4.3 Response of Salinity Parameters to Selected Halophytes Under Salt Stress

The soil pH varied significantly among the plant types and exhibited an increasing trend with increasing salt concentrations. These variations have important ecological implications for halophyte growth and salt tolerance mechanisms. The higher pH values in sea sand compared to UCC soil reflect the alkaline nature of coastal environments where these halophytes naturally occur. Additionally, the higher pH increases with rising salt concentrations, indicating salt‐induced alkalinization, a common phenomenon in saline soils resulting from sodium accumulation and carbonate precipitation (Rengasamy et al. 2022). The ability of *S. portulacastrum* to maintain relatively lower soil pH suggests enhanced rhizosphere acidification (Zhou et al. 2009). This typically occurs through the root exudation of organic acids, which can enhance nutrient availability and promote better salt tolerance.

Similarly, soil electrical conductivity (EC) varied among the plant species but increased exponentially with salt concentrations. This observation reinforces that soil EC is a reliable proxy for soil salt content, as highlighted by Visconti and de Paz (2016). *Paspalum vaginatum* and *S. portulacastrum* and *P. vaginatum* exhibited an interesting phenomenon where soil EC levels were relatively lower despite increasing salt concentrations. This suggests that these halophytes may play a crucial role in reducing soil salinity, potentially through mechanisms such as altering the soil ionic composition or sequestering and taking up salt within plant tissues.

The studied halophytes demonstrated varied responses to increasing salt concentrations and soil types. Particularly, *P. vaginatum* and *S. portulacastrum* actively removed Na^+^ and Cl^−^ from the soil, signifying their extraordinary ability to reduce the accumulation of toxic Na and Cl ions in salt‐affected soils. Furthermore, *S. portulacastrum* and *P. vaginatum* showed phenomenal Na uptake into their leaves, while *S. portulacastrum* and *L. taraxacifolia* showed high leaf Cl uptake. These suggest an increased tolerance to salt stress in these plant species, probably through ion sequestration, antioxidant defense mechanisms, ion homeostasis, and stomatal conductance mechanisms that facilitate osmotic adjustment while mitigating ion toxicity (Bose et al. 2014; Munns 2005; Suriani and Talanca 2018; Gururani et al. 2015). The significant uptake and accumulation of Na and Cl ions in the roots of *S. portulacastrum* and *I. aquatica* further highlight their salt exclusion mechanism, reducing permeability to sodium ions at their root surface. This finding is consistent with the findings of Matsushita and Matoh (1991) and Bhuiyan et al. (2017), who demonstrated increased Na^+^ and Cl^−^ concentrations in the roots of salt‐tolerant reed plants, *Tecticornia pergranulata* and *Thinopyrum ponticum* . These mechanisms enhance plant survival under high salinity and contribute to soil desalination by immobilizing harmful ions within plant tissues.

These results validate the exceptional desalination potential of *P. vaginatum* and *S. portulacastrum* , rendering them suitable for desalination programmes to reclaim saline soils and water. These findings are coherent with the works of Hue et al. (2002), Wang et al. (2022) and Lokhande et al. (2013), which demonstrated the remediation abilities of *P. vaginatum* and *S. portulacastrum* , especially in salt‐affected environments. The special qualities of these halophytes may be attributed to enhanced Ca^2+^ signaling, osmotic adjustments, salt compartmentalisation, as well as other salt tolerance mechanisms, as highlighted by previous studies (Wu et al. 2020; Feng et al. 2014; Peng et al. 2016; Flowers and Colmer 2008).

## 5 Conclusions

Cape Coast's coastal region boasts a diverse array of plant species with multifaceted potential. The identified halophytes in this study are: *Amaranthus* spp., *Canavalia rosea, Capraria biflora, Cyperus ligularis*, *Euphorbia albomarginata, Clitoria ternatea, Furcrea cabuya, Indigofera spicata, Sansevieria masoniana, Ipomoea aquatica, Ipomoea asarifolia, Kalanchoe daigremontiana, Lactuca taraxacifolia, Opuntia* spp., *Paspalum vaginatum, Pedalium murex, Physalis spp*., *Pupalia lappacea, Sesuvium portulacastrum*, and *Cocos nucifera*. These plants offer promising opportunities for various purposes, including human and animal nutrition, ecological restoration, and medicinal and therapeutic applications. For instance, edible species like *Ipomoea aquatica* and *Lactuca taraxacifolia* provide nutrients for humans, while species such as *Sesuvium portulacastrum* and *P. vaginatum* contribute to the stabilization of coastal ecosystems, preventing soil erosion and remediating degraded lands. Conservation efforts and interdisciplinary research are crucial for unlocking the full potential of these botanical resources while ensuring their sustainable use and preservation. The arable soil generally increased the growth of halophytes, revealing the significant role of soil conditions, such as soil structure and chemical and nutrient composition, in enhancing plant growth and development. The growth of *Talinum triangulare* , *I. aquatica* , and *L. taraxacifolia* was hindered at 25 and 50 dS/m salt concentrations, revealing their inability to tolerate and desalinate the soils at these salt concentrations.

On the other hand, *Sesuvium portulacastrum* and *P. vaginatum* demonstrated remarkable phytoremediation capabilities, particularly in mitigating soil salinity and reducing the accumulation of toxic ions such as Na and Cl in the soil. Their effectiveness in reclaiming saline soils, particularly at 25 dS/m salt concentration, highlights their potential for phytoremediation applications, offering sustainable solutions for addressing soil and water salinity issues in affected environments. The potential exists in studying the desalination abilities of *Sesuvium portulacastrum* and *P. vaginatum* in hydroponic systems to assess the possibility of obtaining fresh water from saline water.

## Conflicts of Interest

The authors declare no conflicts of interest.

## Data Availability

All data generated for this study are available within the article.
